# Treatment Strategies for Achieving and Maintaining Low-Risk Status in Pulmonary Arterial Hypertension: A Systematic Review

**DOI:** 10.7759/cureus.118129

**Published:** 2026-10-18

**Authors:** Shahbaz Younas Chaoddhary, Sai Ram Kumar Yatiraju, Aishwarya Chellamani, Eazaz Ali Khan, Farooq Ashraf, Swathi Balasubramanian, Happiness Olaniyi, Harmanpreet Singh, Aliyu O Olaniyi

**Affiliations:** 1 Department of Respiratory Medicine, Stockport NHS Foundation Trust, Stockport, GBR; 2 Department of Surgery, Akhtar Saeed Medical and Dental College, Lahore, PAK; 3 Department of Neonatology, Royal Derby Hospital, Derby, GBR; 4 Department of General Medicine, Stockport NHS Foundation Trust, Stockport, GBR; 5 Department of Medicine for Older People, Stockport NHS Foundation Trust, Stockport, GBR; 6 District Nursing Unit, Stockport NHS Foundation Trust, Stockport, GBR; 7 Department of Stroke Medicine, Stockport NHS Foundation Trust, Stockport, GBR

**Keywords:** combination therapy, low-risk status, prostacyclin, pulmonary arterial hypertension, risk stratification, sotatercept, systematic review, treat-to-target

## Abstract

Achieving and maintaining low-risk status is now the main treatment goal in pulmonary arterial hypertension (PAH), but the evidence connecting individual treatment strategies with low-risk attainment and long-term outcomes is scattered across trials, registries, and cohorts that use different risk tools. We conducted a systematic review following the Preferred Reporting Items for Systematic Reviews and Meta-Analyses (PRISMA) 2020 statement. PubMed, Scopus, Dimensions, and the Cochrane Central Register of Controlled Trials (CENTRAL) were searched from inception to August 27, 2026, for studies of adults with Group 1 PAH that evaluated a treatment strategy and reported a validated risk-stratification outcome. Results were synthesised narratively under the Synthesis Without Meta-analysis (SWiM) guideline, with certainty graded using Grading of Recommendations Assessment, Development and Evaluation (GRADE). Forty-eight reports were included: 10 randomised or prospective interventional studies, 12 secondary analyses of trial data, and 26 observational cohorts. Few patients reached low risk under any strategy, and fewer than half did so after upfront dual oral therapy in most cohorts. Prostacyclin-pathway therapy improved risk status across oral, inhaled, and parenteral routes, although the clearest survival advantage came from parenteral regimens in registry data. Add-on sotatercept improved functional, haemodynamic, and composite clinical outcomes, and these gains were largely sustained at one year. Low-risk status predicted survival, yet it often did not last, and the risk category reached at the first annual review strongly predicted later course. In mediation analyses, change in risk score accounted for only part of the treatment effect, and studies disagreed on whether patients already at low risk benefit from further treatment. Low-risk status is a valuable treatment target and prognostic marker, but it has not been validated as a surrogate endpoint. Early, repeated risk assessment and prompt escalation during the first year after diagnosis are supported by the available evidence.

## Introduction and background

Pulmonary arterial hypertension (PAH) is a rare, progressive disease in which remodelling of the small pulmonary arteries raises pulmonary vascular resistance (PVR) and places a growing load on the right ventricle. When the disease is not controlled, right heart failure and early death follow [1]. Treatment has changed considerably over the past two decades. Sequential monotherapy has largely given way to early combination therapy directed at the endothelin, nitric oxide, and prostacyclin pathways, reflecting the view that PAH involves several dysregulated pathways at once [1,2]. Over the same period, risk stratification has moved to the centre of clinical care. Under a treat-to-target approach, therapy is started and escalated according to how a patient’s risk profile changes over time, with the stated aim of reaching and keeping a low-risk profile [1,3]. The 2022 European Society of Cardiology and European Respiratory Society (ESC/ERS) guidelines formalised this approach, recommending a three-strata model at diagnosis and a simplified four-strata model at follow-up, both combining clinical, functional, biochemical, and, where available, haemodynamic measures [1].

Two factors make the existing evidence difficult to interpret. First, low-risk status is defined inconsistently across studies. The ESC/ERS three- and four-strata models, COMPERA (Comparative, Prospective Registry of Newly Initiated Therapies for Pulmonary Hypertension), COMPERA 2.0, REVEAL 2.0 (Registry to Evaluate Early and Long-Term PAH Disease Management), REVEAL Lite 2, the French non-invasive criteria, and various combinations of individual parameters have all been used [1,4-8]. These frameworks incorporate different variables and thresholds, meaning that the proportion of patients classified as low risk may vary according to the instrument used and the timing of assessment [4,6,7]. These risk-stratification frameworks also differ in their structure and purpose: ESC/ERS and French approaches classify patients using combinations of clinical, functional, biochemical, and, where available, haemodynamic variables, whereas COMPERA and REVEAL are multivariable prediction models that estimate mortality risk. In clinical practice, these tools are used at diagnosis and during follow-up to identify patients at different levels of risk and to guide treatment escalation. Second, uncertainty remains regarding which treatment strategies most reliably achieve and sustain low-risk status. Current approaches include initial monotherapy, upfront dual or triple therapy, sequential escalation, within-pathway switching, prostacyclin-based therapy delivered through different routes, and add-on activin-signalling inhibition with sotatercept for patients who remain at intermediate or high risk [1,9,10]. Treatment may therefore progress from initial monotherapy or upfront combination therapy to sequential escalation, including addition of prostacyclin-pathway agents, within-pathway switching, or addition of newer agents such as sotatercept when treatment targets are not achieved.

Earlier reviews have primarily examined mortality, clinical worsening, exercise capacity, haemodynamics, or the efficacy of individual therapies. A previous systematic review specifically evaluated the prognostic value of serial risk stratification in PAH [11]; therefore, we do not claim that the prognostic importance of serial risk assessment is novel. Our review addresses a complementary question by examining how treatment strategies influence the attainment and maintenance of low-risk status, how findings vary according to the risk-stratification framework used, and whether achieving or maintaining low risk is associated with subsequent clinical outcomes. We also incorporate contemporary evidence on newer treatment approaches, including sequential combination therapy, prostacyclin-based strategies, and activin-signalling inhibition with sotatercept. We therefore addressed three questions: which treatment strategies are associated with achieving low-risk status; how durable is low-risk status once achieved; and is achieving or maintaining low-risk status associated with better clinical outcomes?

## Review

Methodology

Protocol and Eligibility Criteria

The review was conducted and reported according to the Preferred Reporting Items for Systematic Reviews and Meta-Analyses (PRISMA) 2020 statement [12]. Eligibility criteria, search strategy, and synthesis methods were set before title and abstract screening began. A written protocol was prepared in advance but was not prospectively registered, and deviations from it are reported where they occurred. Eligibility was framed using population, intervention, comparator, outcome, and study design. We included studies of adults aged 18 years or older with Group 1 PAH, covering idiopathic, heritable, drug- and toxin-associated, connective tissue disease-associated (CTD-PAH), human immunodeficiency virus (HIV)-associated, porto-pulmonary, congenital heart disease-associated (CHD-PAH), and other recognised Group 1 subtypes. Diagnosis by right heart catheterisation was required where reported, using the criteria in use at the time of each study. Studies of mixed pulmonary hypertension populations were eligible only if Group 1 data could be separated. We excluded studies focused on pulmonary hypertension due to left heart disease, lung disease or hypoxia, or chronic thromboembolic disease, as well as paediatric-only, animal, and in-vitro studies.

Eligible interventions were PAH-targeted drug strategies intended to improve clinical status or reduce risk. These were grouped in advance as initial strategies (monotherapy, upfront dual therapy, upfront triple therapy), sequential escalation (including addition of prostacyclin-pathway therapy), switching within a pathway, goal-oriented or treat-to-target management, and add-on therapy with newer agents. Comparators could be placebo, standard care, another regimen, escalation versus no escalation, or, for single-arm studies, the pre-treatment state. Randomised controlled trials (RCTs), prospective interventional and open-label extension studies, secondary analyses of trial data, and prospective or retrospective cohort and registry studies were eligible. Case reports, case series, reviews, editorials, letters without extractable data, and conference abstracts with insufficient outcome data were excluded, although reviews and guidelines were used for citation searching. Only English-language publications were included.

The primary outcome was formal attainment of low-risk status at follow-up using a validated multidimensional tool, such as the ESC/ERS three- or four-strata models, COMPERA, COMPERA 2.0, REVEAL 2.0, REVEAL Lite 2, or the French non-invasive criteria. To keep different kinds of evidence apart, outcomes were placed in three categories. Category 1 was formal low-risk attainment on a validated tool, which counted as direct evidence for the primary outcome. Category 2 was an improvement in the risk score or category that fell short of low risk. Category 3 was attainment of a single treatment target, such as a six-minute walk distance (6MWD) threshold. The phase 4 COMPASS-3 study, for example, used a 6MWD target and was therefore treated as Category 3 evidence. Secondary outcomes were maintenance of low risk, change in risk category, time to low risk, mortality, clinical worsening, hospitalisation, transplantation, 6MWD, World Health Organization functional class (WHO-FC), quality of life, activity measures, natriuretic peptides, haemodynamic and imaging measures, and safety.

During full-text assessment, we changed how one group of studies was handled. Studies that only derived or externally validated a risk-prediction tool, without evaluating a treatment strategy, did not meet the intervention criterion. The REVEAL 2.0, REVEAL Lite 2, and COMPERA 2.0 derivation studies, the French four-strata validation, the updated Swedish Pulmonary Arterial Hypertension Registry (SPAHR) calculator, and the Pulmonary Hypertension Society of Australia and New Zealand validation were therefore cited as background for the comparison of risk tools and were not counted as included studies [4-8,13].

Search Strategy and Study Selection

PubMed, Dimensions, Scopus, and the Cochrane Central Register of Controlled Trials (CENTRAL) were searched from inception to August 27, 2026. Each database strategy combined three concept groups using the AND operator: terms for PAH and its subtypes; terms for risk stratification, including established risk tools and their component measures; and terms for treatment, including combination and sequential therapy and the principal PAH-targeted drug classes. We did not rely on the phrase “low-risk status", because relevant studies may report named risk scores or their individual components without using this terminology. The database-specific search strategies are provided in Tables 1-4. The Dimensions search was conducted using the platform’s Full search function. The search strategy followed the same conceptual structure as PubMed, using three Boolean concept blocks covering pulmonary arterial hypertension, risk stratification, and treatment strategies. The same term lists were used, with synonyms combined using OR and the three concept blocks combined using AND. Database-specific search approaches are described in Tables 1-4.

**Table 1:** PubMed search strategy (inception to August 27, 2026)

Step	Search strategy
#1	"pulmonary arterial hypertension"[tiab] OR "PAH"[tiab] OR "idiopathic pulmonary arterial hypertension"[tiab] OR "heritable pulmonary arterial hypertension"[tiab] OR "connective tissue disease-associated pulmonary hypertension"[tiab] OR "portopulmonary hypertension"[tiab] OR "congenital heart disease-associated pulmonary hypertension"[tiab] OR "HIV-associated pulmonary hypertension"[tiab] OR "Group 1 pulmonary hypertension"[tiab] OR "Hypertension, Pulmonary"[Mesh]
#2	"risk stratification"[tiab] OR "risk assessment"[tiab] OR "low risk"[tiab] OR "risk score"[tiab] OR "risk category"[tiab] OR "treat-to-target"[tiab] OR "treatment goal"[tiab] OR "COMPERA"[tiab] OR "REVEAL"[tiab] OR "REVEAL Lite"[tiab] OR "French risk criteria"[tiab] OR "functional class"[tiab] OR "6-minute walk"[tiab] OR "six-minute walk"[tiab] OR "BNP"[tiab] OR "NT-proBNP"[tiab] OR "brain natriuretic peptide"[tiab]
#3	"combination therapy"[tiab] OR "dual therapy"[tiab] OR "triple therapy"[tiab] OR "upfront therapy"[tiab] OR "sequential therapy"[tiab] OR "treatment escalation"[tiab] OR "treatment intensification"[tiab] OR "prostacyclin"[tiab] OR "endothelin receptor antagonist"[tiab] OR "phosphodiesterase-5 inhibitor"[tiab] OR "soluble guanylate cyclase stimulator"[tiab] OR "prostacyclin receptor agonist"[tiab] OR "selexipag"[tiab] OR "treprostinil"[tiab] OR "iloprost"[tiab] OR "epoprostenol"[tiab] OR "macitentan"[tiab] OR "ambrisentan"[tiab] OR "bosentan"[tiab] OR "sildenafil"[tiab] OR "tadalafil"[tiab] OR "riociguat"[tiab] OR "sotatercept"[tiab]
#4	#1 AND #2 AND #3
#5	#4 AND English[Language]
	Total records retrieved: 769

**Table 2:** Scopus search strategy (inception to August 27, 2026)

Step	Search strategy
Population block	TITLE-ABS-KEY("pulmonary arterial hypertension" OR "idiopathic pulmonary arterial hypertension" OR "heritable pulmonary arterial hypertension" OR "connective tissue disease-associated pulmonary hypertension" OR "portopulmonary hypertension" OR "congenital heart disease-associated pulmonary hypertension" OR "HIV-associated pulmonary hypertension" OR "Group 1 pulmonary hypertension")
Risk block	TITLE-ABS-KEY("risk stratification" OR "risk assessment" OR "low risk" OR "risk score" OR "treat-to-target" OR "treatment goal" OR "COMPERA" OR "REVEAL" OR "REVEAL Lite" OR "French risk criteria" OR "functional class" OR "6-minute walk" OR "BNP" OR "NT-proBNP")
Treatment block	TITLE-ABS-KEY("combination therapy" OR "dual therapy" OR "triple therapy" OR "upfront therapy" OR "sequential therapy" OR "treatment escalation" OR "prostacyclin" OR "endothelin receptor antagonist" OR "phosphodiesterase-5 inhibitor" OR "soluble guanylate cyclase stimulator" OR "prostacyclin receptor agonist" OR "selexipag" OR "treprostinil" OR "macitentan" OR "ambrisentan" OR "bosentan" OR "sildenafil" OR "tadalafil" OR "riociguat" OR "sotatercept")
Language limit	AND LIMIT-TO(LANGUAGE, "English")
	Total records retrieved: 1,839

**Table 3:** Dimensions search strategy (inception to August 27, 2026)

Step	Search strategy
Full search	Free-text 'Full search' function using Boolean operators. The three concept blocks (population, risk stratification, treatment) were combined as in PubMed, using the same term lists.
Filters	English language and article/review publication types. Exact query strings in Dimensions differ by session and are captured in the study team's search log archived with the study protocol.
	Total records retrieved: 1,217

**Table 4:** Cochrane CENTRAL (August 27, 2026) CENTRAL: Cochrane Central Register of Controlled Trials

Step	Search strategy
#1	MeSH descriptor: [Hypertension, Pulmonary] explode all trees
#2	("pulmonary arterial hypertension" OR "idiopathic pulmonary arterial hypertension" OR "heritable pulmonary arterial hypertension" OR "connective tissue disease-associated pulmonary hypertension" OR "portopulmonary hypertension" OR "congenital heart disease-associated pulmonary hypertension" OR "HIV-associated pulmonary hypertension" OR "Group 1 pulmonary hypertension"):ti,ab,kw
#3	#1 OR #2
#4	("risk stratification" OR "risk assessment" OR "low risk" OR "risk score" OR "treat-to-target" OR "COMPERA" OR "REVEAL" OR "REVEAL Lite" OR "French risk criteria" OR "functional class" OR "6-minute walk" OR "six-minute walk" OR "BNP" OR "NT-proBNP"):ti,ab,kw
#5	("combination therapy" OR "dual therapy" OR "triple therapy" OR "upfront therapy" OR "sequential therapy" OR "treatment escalation" OR "prostacyclin" OR "endothelin receptor antagonist" OR "phosphodiesterase-5 inhibitor" OR "soluble guanylate cyclase stimulator" OR "prostacyclin receptor agonist" OR "selexipag" OR "treprostinil" OR "macitentan" OR "ambrisentan" OR "bosentan" OR "sildenafil" OR "tadalafil" OR "riociguat" OR "sotatercept"):ti,ab,kw
#6	#3 AND #4 AND #5 [limited to Trials]
	Total records retrieved: 1,791

The searches identified 5,616 records, comprising 769 from PubMed, 1,217 from Dimensions, 1,839 from Scopus, and 1,791 from CENTRAL. After removal of 1,419 duplicate records, 4,197 records underwent title and abstract screening, of which 3,447 were excluded. The remaining 750 reports underwent full-text assessment for eligibility. At full-text assessment, 696 reports were excluded for predefined reasons, including failure to evaluate a treatment strategy, absence of a validated risk-stratification outcome, ineligible publication type or insufficient extractable data, ineligible or non-separable population, overlapping cohorts without a new outcome or follow-up, and non-English publication. In addition, six risk-tool development or validation reports were excluded from the evidence synthesis because they did not evaluate a treatment strategy; these reports were retained as background references to inform the description and comparison of risk-stratification instruments. Forty-eight reports met all eligibility criteria and were included in the review. The study-selection process is presented in Figure 1.

**Figure 1:**
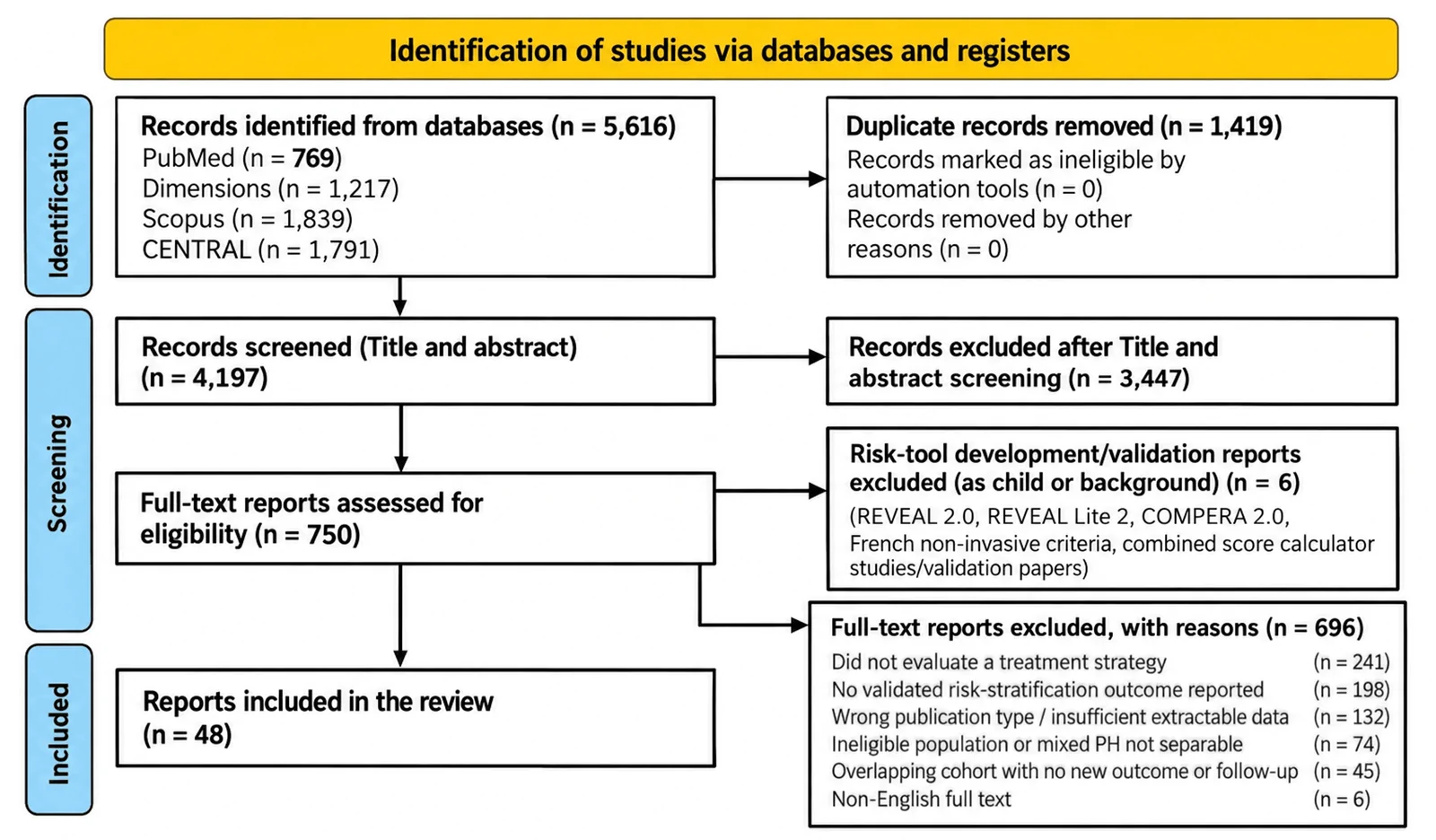
PRISMA 2020 flow diagram for the study selection process PRISMA: Preferred Reporting Items for Systematic Reviews and Meta-Analyses; CENTRAL: Cochrane Central Register of Controlled Trials; COMPERA: Comparative, Prospective Registry of Newly Initiated Therapies for Pulmonary Hypertension; REVEAL: Registry to Evaluate Early and Long-Term Pulmonary Arterial Hypertension Disease Management

Two reviewers independently screened records at each stage, with disagreements resolved through discussion or, when necessary, adjudication by a third reviewer. Because individual PAH trials and registries may generate multiple publications from the same underlying population, we identified the parent study or cohort for each report and retained additional publications only when they provided new outcomes, longer follow-up, or a distinct analytic question. Overlapping reports were therefore treated as a single body of evidence when estimating findings from the same underlying population. 

Data Extraction and Risk of Bias

Two reviewers extracted data independently using a piloted form, resolving disagreements by consensus or third-reviewer adjudication. We recorded study design, setting, recruitment period, sample size, follow-up, and funding; patient age, sex, PAH subtype, baseline WHO-FC, 6MWD, natriuretic peptide levels, haemodynamics, baseline risk, and comorbidities; the drugs used, their sequence, route, dose, and discontinuation; the risk tool, its variables, the definition of low risk, and the timing of assessments; and clinical, functional, haemodynamic, and safety outcomes.

Risk of bias was assessed independently by two reviewers. We used the Cochrane Risk of Bias 2 (RoB 2) tool for randomised trials [14], the Risk Of Bias In Non-randomised Studies of Interventions (ROBINS-I) tool for non-randomised comparisons [15], and the Quality In Prognosis Studies (QUIPS) tool for studies mainly concerned with prognostic factors for low-risk attainment [16]. Single-arm studies were assessed with ROBINS-I using the pre-treatment state as the comparator, recognising that the lack of a control group limits causal conclusions. Judgements informed interpretation and sensitivity analyses and were not converted into a numerical score. Publication and reporting bias were considered narratively. We did not systematically compare trial registrations with published reports, and funnel plots were not drawn because no single comparison had enough studies sharing an effect measure. 

Data Synthesis

Because studies differed widely in treatment strategy, population, follow-up duration, and risk-stratification tools, we conducted a structured narrative synthesis in accordance with the Synthesis Without Meta-analysis (SWiM) guideline [17]. Differences in baseline risk, treatment exposure, outcome definitions, assessment timing, and risk-stratification frameworks were considered likely to affect the magnitude and clinical interpretation of treatment-associated changes. Accordingly, studies were not assumed to be directly comparable, and quantitative synthesis was considered only where clinical and methodological comparability was sufficient.

Pooling was considered for four potentially comparable subsets: sotatercept versus placebo, low-risk attainment with oral prostacyclin-pathway therapy, risk-category change with riociguat, and clinical worsening. None met the prespecified threshold of at least three sufficiently comparable studies reporting the same effect measure for the same comparison. For example, the sotatercept trials differed in baseline risk and composite outcome definitions, while the riociguat analyses included multiple reports derived from the same parent trials. We therefore report individual study estimates, including proportions, hazard ratios (HRs), odds ratios (ORs), and mean differences, as reported in the original studies, with 95% confidence intervals where available.

Overlapping reports were considered together when interpreting findings, and multiple analyses of the same underlying population were not treated as independent replications. Conflicting findings were examined in relation to study design, population, and risk of bias; vote-counting was not used. Results are organised by treatment strategy, followed by comparisons of risk-stratification tools, subgroup findings by baseline risk, phenotype, comorbidity and age, follow-up duration, and sensitivity analyses.

Meta-regression was not undertaken because the number of sufficiently comparable independent studies was inadequate for reliable modelling, and substantial clinical and methodological heterogeneity across populations, treatment strategies, outcome definitions, risk-stratification tools, and follow-up periods would have limited the interpretability of between-study associations. Certainty of evidence for the main outcomes was assessed using Grading of Recommendations Assessment, Development and Evaluation (GRADE) [18]. ROBINS-I assessments of registry-based and observational studies are provided in Appendix 5.

Results

The 48 included reports comprised 10 randomised or prospective interventional studies, 12 secondary or post hoc analyses of trial data, and 26 registry or observational cohorts (Table 5) [2,3,9,10,19-62]. They do not represent 48 separate populations. Several came from the same trial programmes (AMBITION, GRIPHON, PATENT, TRITON, STELLAR, and FREEDOM) or the same registries and networks (the French Pulmonary Hypertension Registry, SPAHR, COMPERA, and Italian and Chinese multicentre groups). Sample sizes ranged from 25 patients in a single-centre Mexican cohort [19] to 2,031 in the Chinese national registry [20], and follow-up ranged from 12 weeks in trial analyses to 10 years in registry cohorts. Most patients had idiopathic, heritable, or drug-associated PAH. Separate evidence was available for CTD-PAH [21], CHD-PAH [20,22], portopulmonary hypertension [23], and HIV-associated PAH [24]. Most studies came from Europe, North America, and East Asia, with one cohort each from Latin America and Africa.

**Table 5:** Characteristics of the 48 included reports, with underlying study or cohort and outcome contribution 6MWD: six-minute walk distance; CHD-PAH: congenital heart disease-associated pulmonary arterial hypertension; COMPERA: Comparative, Prospective Registry of Newly Initiated Therapies for Pulmonary Hypertension; CTD-PAH: connective tissue disease-associated pulmonary arterial hypertension; CWFS: clinical worsening-free survival; ERA: endothelin receptor antagonist; ESC/ERS: European Society of Cardiology/European Respiratory Society; iPHNET: Italian Pulmonary Hypertension Network; NT-proBNP: N-terminal pro-B-type natriuretic peptide; PAH: pulmonary arterial hypertension; PDE5i: phosphodiesterase type-5 inhibitor; PHORA: Pulmonary Hypertension Outcomes Risk Assessment; PVR: pulmonary vascular resistance; RCT: randomised controlled trial; REVEAL: Registry to Evaluate Early and Long-Term Pulmonary Arterial Hypertension Disease Management; RHC: right heart catheterisation; RV: right ventricular; SPAHR: Swedish Pulmonary Arterial Hypertension Registry; WHO-FC: World Health Organization functional class

Report	Design	Underlying study/cohort (n)	Treatment strategy or focus	Risk framework	Contribution to this review
Galiè 2015 (AMBITION) [2]	RCT	Multinational (500)	Upfront ambrisentan + tadalafil vs pooled monotherapy	Not a primary endpoint	Parent trial for upfront dual therapy; parent of Fauvel 2023
Fauvel 2023 [25]	Post hoc of RCT	AMBITION (500)	Upfront dual vs monotherapy by baseline risk	REVEAL 2.0; PHORA	Only formal test of treatment benefit within a validated low-risk subgroup
Sitbon 2015 (GRIPHON) [26]	RCT	Multinational (1,156)	Selexipag add-on to background therapy	Not a primary endpoint	Parent trial for oral prostacyclin-pathway add-on
Sitbon 2020 [27]	Post hoc of RCT	GRIPHON (1,156)	Selexipag by number of low-risk criteria and REVEAL 2.0	French non-invasive; REVEAL 2.0	Treatment-induced change in low-risk criteria count
Benza 2024 (GRIPHON) [28]	Post hoc of RCT	GRIPHON (1,156)	Selexipag, change in REVEAL Lite 2 risk status	REVEAL Lite 2	Mediation analysis of risk improvement as explanatory pathway
Chin 2021 (TRITON) [29]	RCT	Multinational (247)	Upfront oral triple vs upfront dual therapy	Not a primary endpoint	Only randomised comparison of initial triple vs dual oral therapy
McLaughlin 2024 [30]	Pooled post hoc of RCTs	TRITON + REPAIR (148)	Initial macitentan + tadalafil, with vs without cardiac comorbidities	Four-strata; REVEAL Lite 2	Risk-status change after upfront dual therapy by comorbidity status
Hoeper 2023 (STELLAR) [9]	RCT	Multinational (323)	Sotatercept add-on to background dual/triple therapy	Not a primary endpoint	Parent activin-signalling trial
Souza 2023 [31]	Haemodynamic sub-study of RCT	STELLAR (298 RHC; 275 echo)	Sotatercept add-on	Haemodynamic outcomes	Haemodynamic and right heart function effects
Humbert 2025 (ZENITH) [10]	RCT	Multinational (172)	Sotatercept add-on in high-risk PAH	REVEAL Lite 2 at entry	Risk-score-defined high-risk population; composite clinical outcome
Preston 2025 (SOTERIA) [32]	Open-label extension	Prior sotatercept studies (426)	Long-term sotatercept add-on	Simplified French risk score	Durability of risk-score improvement to one year
Benza 2018 (COMPASS-3) [33]	Phase 4 prospective single-arm	United States, 23 centres (100)	Bosentan monotherapy with protocol-directed escalation	Goal-directed 6MWD threshold	Category 3 evidence on monotherapy goal attainment
Hoeper 2017 (RESPITE) [34]	Prospective open-label single-arm	Multinational (61)	Switch from PDE5i to riociguat	WHO-FC, 6MWD, NT-proBNP targets	Within-pathway switching in insufficient responders
Benza 2022 (PATENT) [35]	Post hoc of RCT	PATENT-1/PATENT-2	Riociguat, change in REVEAL Lite 2	REVEAL Lite 2	Risk-score change and its association with survival and CWFS
Hoeper 2025 (PATENT) [36]	Post hoc of RCT	PATENT-1/PATENT-2 (314 at baseline)	Riociguat, four-strata application	COMPERA 2.0	Risk-category improvement by intermediate substratum
Simonneau 2020 [37]	Post hoc of RCT	PATENT-1/PATENT-2	Riociguat, REPLACE composite endpoint applied	REPLACE composite (Category 2/3)	Composite clinical improvement and its prognostic value
Rosenkranz 2025 [38]	Pooled post hoc of RCTs	PATENT-1/2, PATENT PLUS, REPLACE	Riociguat by cardiometabolic comorbidity count	COMPERA 2.0	Risk-status response by comorbidity burden
Benza 2022 (FREEDOM-EV) [39]	Post hoc of RCT	FREEDOM-EV (690)	Oral treprostinil add-on to oral monotherapy	French non-invasive; REVEAL 2.0/Lite 2	Therapy-responsiveness of contemporary risk scores
Lachant 2026 [40]	Pooled analysis of trials and chart review	FREEDOM-C, FREEDOM-C2, chart review	Oral treprostinil on background mono- vs dual therapy	Risk and functional measures	Effect of background regimen on add-on response
Tonelli 2020 [41]	Post hoc of RCTs	TRIUMPH (148); BEAT (73)	Inhaled treprostinil add-on	French non-invasive; REVEAL 2.0	Risk-stratum improvement with inhaled prostacyclin
Miller 2023 (EXPEDITE) [42]	Prospective single-arm	Multicentre (29)	Parenteral treprostinil induction then oral treprostinil	REVEAL Lite 2	Rapid-induction strategy and risk-score change
Wang 2023 [43]	Prospective multicentre trial	China (83)	Ambrisentan, naive vs add-on	Risk improvement; time to clinical improvement	Time-to-attainment as an endpoint construct
Boucly 2021 [44]	Retrospective registry cohort	French PH Registry (1,611)	Initial mono-, dual-, or parenteral-prostacyclin-containing triple therapy	ESC/ERS-based	Principal long-term survival evidence by initial strategy
Badagliacca 2021 [45]	Prospective multicentre cohort	Italy (181)	Upfront ERA + PDE5i	ESC/ERS; REVEAL	Low-risk attainment at 6 months and its haemodynamic determinants
Badagliacca 2022 [46]	Prospective multicentre cohort	Italy, iPHNET (181)	Upfront oral combination by cardiac comorbidity count	ESC/ERS; REVEAL 2.0	Comorbidity-related attenuation of PVR and risk response
D’Alto 2020 [47]	Retrospective multicentre cohort	Italy (106)	Upfront ambrisentan + tadalafil, 2-year follow-up	REVEAL 2.0	Medium-term durability of risk improvement
Badagliacca 2018 [48]	Retrospective multicentre cohort	Italy (69 plus 2 matched cohorts)	Upfront combination vs monotherapy, right ventricular outcomes	ESC/ERS-based; RV imaging	Right ventricular remodelling by strategy
Distler 2024 [21]	Registry cohort	COMPERA, CTD-PAH (607)	Initial ERA + PDE5i in CTD-PAH	COMPERA four-strata	Phenotype-specific survival by initial strategy
Rosenkranz 2023 [49]	Registry cohort	COMPERA (1,120)	Comorbidity subgroup analysis	COMPERA four-strata	Treatment response and prognostic validity with comorbidities
Kylhammar 2018 [3]	Registry cohort	SPAHR (530)	Treat-to-target management	SPAHR/ESC-ERS	Foundational evidence that early low-risk status predicts survival
Bouzina 2021 [50]	Registry cohort	SPAHR (503)	Longitudinal risk-guided follow-up	SPAHR/ESC-ERS	Risk-category transitions and durability over three years
Quan 2022 [20]	National prospective registry	China (2,031)	Goal-oriented treatment in real-world practice	ESC/ERS-derived; REVEAL	Applicability of risk frameworks and goal-oriented care outside high-income Western settings
Pentikäinen 2025 (FINPAH) [51]	National cohort	Finland (268 PAH)	Real-world initiation and escalation patterns	COMPERA 2.0	Guideline concordance and low-risk attainment at one year
Cui 2026 [52]	Retrospective cohort	China, single centre (153/146)	Upfront vs sequential triple therapy with selexipag	French non-invasive	Direct comparison of upfront vs sequential triple therapy
Zhao 2024 [53]	Multicentre retrospective cohort	China, 10 centres (192)	Oral sequential triple therapy with selexipag	Low-risk criteria count	Real-world sequential oral triple therapy
Tokgöz 2026 [54]	Single-centre registry cohort	Türkiye (127 of 1,160)	Sequential triple therapy with selexipag, by dose	COMPERA 2.0; ESC/ERS; REVEAL Lite 2	Durability of early risk improvement beyond one year
Deshwal 2023 [55]	Retrospective cohort	United States, single centre (55 of 414)	Add-on sequential triple combination therapy	REVEAL 2.0; four-strata	Long-term risk reduction and predictors in a diverse population
Bravo-Marqués 2024 (RAMPHA) [56]	Retrospective multicentre cohort	Spain, Andalusia (42)	Selexipag add-on in routine care	ESC/ERS-based risk profile	Real-world magnitude and limits of risk-profile change
D’Alto 2026 (INSPECTIO) [57]	Prospective multicentre observational	Italy, 29 centres	Macitentan and/or selexipag within combination therapy	Non-invasive low-risk criteria; REVEAL	Twelve-month change in low-risk criteria in routine care
Cortes-Telles 2026 [19]	Retrospective cohort	Mexico, single centre (25)	Sequential add-on ERA + PDE5i in Group 1.4 PAH	COMPERA 2.0; REVEAL Lite 2	Risk reclassification in an under-represented region and phenotype
Escribano-Subias 2020 (PRACMA) [58]	Retrospective observational	Spain (81; 57 scored)	Macitentan, incident and prevalent patients	REVEAL	Risk-score change with ERA initiation in routine care
Vaidya 2024 [59]	Retrospective chart review and survey	United States (350 records)	Parenteral prostacyclin in intermediate-risk patients	COMPERA 2.0	Implementation gap between guideline advice and practice
Gomez Rojas 2025 [60]	Single-centre observational cohort	United States (45)	Sotatercept in patients with cardiopulmonary comorbidities	REVEAL Lite 2	Real-world extension of trial evidence to excluded comorbid patients
Lachant 2023 [61]	Prospective single-centre observational	United States (30 paired)	Actigraphy after treatment intensification	Activity-based measures (Category 3)	Novel functional outcome sensitive to escalation
Enderby 2018 [62]	Retrospective single-institution review	United States (single centre)	Inhaled treprostinil in Group 1 PAH	Functional and tolerability measures	Tolerability and clinical efficacy of inhaled prostacyclin in routine care
Kiko 2024 [23]	Retrospective cohort	Japan, single centre (33)	Upfront combination vs monotherapy in portopulmonary hypertension	Not applicable (phenotype cohort)	Phenotype-specific haemodynamic and event outcomes
Yaylalı 2023 [22]	Retrospective multicentre cohort	Türkiye (126)	Phenotype-specific CHD-PAH cohort	French non-invasive; REVEAL Lite 2	Feasibility but not validity of standard tools in CHD-PAH
Thienemann 2022 (PAPUCO) [24]	Registry cohort	Pan-African, 9 centres (134)	Phenotype-specific HIV-associated cohort	Not applicable (phenotype cohort)	Only African evidence; distinct high-risk phenotype

Overlapping reports were handled as follows. The GRIPHON trial [26] and its two risk-based re-analyses [27,28] were treated as one population, with treatment-effect estimates taken from the parent trial. The three PATENT re-analyses [35-37] and the pooled riociguat analysis [38] were treated as one body of evidence, so agreement between them was not counted as independent replication. The two SPAHR reports [3,50] address different questions and were retained separately. STELLAR [9], its haemodynamic sub-study [31], and the SOTERIA extension [32] form one lineage. The Italian reports by Badagliacca and colleagues [45,46,48] draw on an overlapping national network, and the 181-patient upfront-combination cohort was counted once when estimating low-risk attainment.

Monotherapy and Goal-Directed Escalation

Monotherapy rarely produced a lasting low-risk profile. In COMPASS-3, only 17% of patients reached the 6MWD target on bosentan alone at 16 weeks; most needed sildenafil added, and a further 20% reached the target by week 28 [33]. In the Spanish PRACMA cohort, starting macitentan in routine care was followed by a mean fall of 1.4 points in REVEAL score over a median of about 10 months [58]. A prospective Chinese ambrisentan trial reported risk improvement in 64.6% of patients within 24 weeks, with faster improvement in treatment-naive than in add-on patients [43]. In Finland, where 52% of patients started on monotherapy, 35% were low-risk at one year, rising to 44% among those diagnosed after 2015 as dual therapy became more common [51]. Across these studies, most patients did not reach their goal on a single drug.

Upfront Dual Combination Therapy

AMBITION showed that starting ambrisentan and tadalafil together halved the risk of clinical failure compared with either drug alone, mainly through fewer PAH-related hospitalisations [2]. Reaching low risk after upfront dual therapy was still uncommon. In an Italian prospective cohort given an endothelin receptor antagonist (ERA) with a phosphodiesterase type-5 inhibitor (PDE5i), 34.8% of patients were low-risk at six months by REVEAL and 43.1% by the ESC/ERS approach, even though functional class and 6MWD improved, and the chance of reaching low risk was closely tied to the size of the haemodynamic response [45]. After two years of upfront ambrisentan and tadalafil in a related cohort, 43% had a low REVEAL 2.0 score, compared with 9% at baseline, but 57% remained at intermediate or high risk despite a mean 37% fall in PVR [47]. In a pooled analysis of the TRITON and REPAIR populations, initial macitentan and tadalafil lowered PVR by 55% in patients without cardiac comorbidities and by 50% in those with one or two, and most patients in both groups improved their risk status at week 26 [30]. The Italian comorbidity analysis was less reassuring, showing median PVR reductions of 45.0%, 30.3%, and 24.3% in patients with none, one, and two or more cardiac comorbidities [46]. In the French registry, initial dual therapy gave no overall survival advantage over monotherapy, with five-year survival of about 61% in both groups, although benefit was seen in patients at intermediate risk at diagnosis [44]. In a COMPERA analysis of CTD-PAH, initial ERA and PDE5i therapy was associated with better survival than monotherapy in systemic sclerosis-associated PAH, the subgroup with the worst prognosis [21].

Sequential Escalation and Switching Within a Pathway

In a Chinese real-world cohort, upfront triple therapy including selexipag resulted in a greater proportion of patients achieving all three French non-invasive low-risk criteria than sequentially adding selexipag to dual therapy (81.5% versus 44.3%). Upfront triple therapy was also associated with a lower risk of disease progression (HR 0.177, 95% CI 0.035-0.898), although the authors considered this comparison exploratory because it was based on only six events [52]. A 10-centre Chinese cohort of 192 patients receiving selexipag added to an ERA and either a PDE5i or riociguat provided further real-world evidence supporting the feasibility of sequential oral triple therapy over six months [53].

In a United States cohort, the proportion of patients classified as low risk increased from 38.2% to 61.8% over a median of 68 weeks after a third drug was added to dual therapy in patients who had not achieved treatment goals; no patient remained at high risk [55]. However, the retrospective, single-centre design and absence of a concurrent control group limit causal interpretation. These changes cannot be attributed to treatment alone and should be regarded as supportive observational evidence rather than definitive evidence of treatment efficacy.

Other cohorts reported more modest or less sustained improvements. A Turkish registry of 127 patients receiving sequential triple therapy with selexipag found early improvements in risk classification using COMPERA 2.0, ESC/ERS, and REVEAL Lite 2, but suggested that these improvements might wane after the first year; early reclassification also showed limited ability to discriminate long-term survival [54]. In the Spanish RAMPHA cohort of 42 patients, adding selexipag tripled the proportion classified as low risk at six to twelve months, although the overall shift did not reach statistical significance [56]. Similarly, in the 29-centre Italian INSPECTIO study, the mean number of non-invasive low-risk criteria changed little over 12 months among patients receiving macitentan, selexipag, or both [57].

Switching and soluble guanylate cyclase stimulation were studied in one open-label trial and in re-analyses of PATENT. In RESPITE, switching from a PDE5i to riociguat in patients with an inadequate response increased 6MWD by a mean of 31 m, lowered N-terminal pro-B-type natriuretic peptide (NT-proBNP) by a mean of 347 pg/mL, and improved functional class in 54% of patients at 24 weeks, with clinical worsening in 10% [34]. In PATENT, riociguat improved REVEAL Lite 2 score and risk stratum more often than placebo, and both the baseline score and its change were associated with survival and clinical worsening-free survival [35]. Applying COMPERA 2.0 to the same trials, riociguat improved risk category more often than placebo in both intermediate-low (38% versus 22%) and intermediate-high (42% versus 31%) patients, and risk strata at baseline and week 12 separated two-year survival and clinical worsening [36]. Patients in PATENT who met the REPLACE composite improvement endpoint also had lower later rates of death and clinical worsening [37].

Triple Therapy

TRITON is the only trial to randomise patients to initial triple versus initial dual oral therapy. It did not meet its primary endpoint, as PVR fell by 54% with triple therapy and 52% with dual therapy at 26 weeks [29]. Exploratory analyses suggested less disease progression (HR 0.59, 95% CI 0.32 to 1.09) and lower mortality (HR 0.23, 95% CI 0.05 to 1.04) with triple therapy, but the trial was not designed or powered for these outcomes, and the authors presented them as hypothesis-generating [29]. These results apply to oral regimens and should not be extended to triple therapy that includes a parenteral prostacyclin. Registry data for parenteral regimens provide additional evidence. In the French Pulmonary Hypertension Registry, patients receiving initial triple-combination therapy, including a parenteral prostacyclin, had higher five-year survival than those receiving monotherapy or dual-combination therapy (91% vs. 61%); transplant-free survival was 75% with triple therapy compared with 56% with dual therapy and 58% with monotherapy. Among patients at intermediate risk, initial triple therapy was independently associated with lower mortality (HR 0.29, 95% CI 0.11 to 0.80) [44]. The authors also reported that, after a median follow-up of five months, 78% of patients receiving initial triple therapy had achieved three or four low-risk criteria, compared with 47% receiving dual therapy and 36% receiving monotherapy. However, these reported percentages are not reproduced exactly by the distribution shown in the original article; consequently, the low-risk-criteria attainment estimates were interpreted cautiously and were not used as definitive quantitative estimates in the synthesis [44]. In 33 patients with porto-pulmonary hypertension, upfront combination therapy reduced PVR more than monotherapy (57% versus 32%, p=0.006) and improved event-free survival (p=0.016), with no detectable difference in mortality [23]. The EXPEDITE study tested a short course of parenteral treprostinil followed by transition to the oral form, reaching a mean oral dose of 16.4 mg per day by week 16. Of the patients, 79% reached the target dose, and the median REVEAL Lite 2 score fell from 6 to 3.5 [42]. A United States chart review showed how late parenteral therapy is often started in practice: among 280 patients with COMPERA 2.0 data at initiation, 62% were intermediate-high risk, 33% intermediate-low, and 3% low risk [59].

Prostacyclin-Pathway Therapy by Route

In GRIPHON, adding selexipag to background therapy lowered the risk of a first morbidity or mortality event by 40% (HR 0.60, 99% CI 0.46 to 0.78), mainly through less disease progression and hospitalisation, and the effect was similar regardless of background regimen [26]. Two re-analyses looked at risk status. The first showed that both the number of low-risk criteria and REVEAL 2.0 category predicted outcome at every time point, that patients meeting all three low-risk criteria at baseline had a 94% lower event risk than those meeting none, and that selexipag-treated patients were more likely to gain low-risk criteria by week 26 (OR 1.69, p=0.0002) [27]. The second, using REVEAL Lite 2, found fewer events with selexipag in every baseline risk category and odds of risk improvement of roughly 1.8 to 2.0 at weeks 16, 26, and 52. In its mediation analysis, improvement in risk status at week 16 accounted for 19.1% of the treatment effect overall and 47.0% in patients with a baseline score of 7 or more [28]. Oral treprostinil showed a similar pattern. In FREEDOM-EV, current risk scores predicted clinical worsening and responded to treatment [39], and pooled FREEDOM-C and FREEDOM-C2 data showed that the number of background drugs did not change the achieved dose or the functional response [40]. For inhaled treprostinil, a post hoc analysis of TRIUMPH and BEAT found improvement in REVEAL 2.0 risk stratum at week 12 compared with placebo (OR 2.38, 95% CI 1.09 to 5.19), and both the French approach and REVEAL 2.0 predicted later survival, clinical worsening, and hospitalisation [41]. A single-centre review also described tolerability and outcomes with inhaled treprostinil in routine care [62]. Considered alongside the triple-therapy data, these findings suggest that the survival benefit of prostacyclin-pathway escalation is clearest with parenteral treatment, while oral and inhaled agents have stronger evidence for improving risk status and slowing progression than for reducing mortality over the follow-up periods studied.

Treat-to-Target Management

Two SPAHR studies underpin the treat-to-target approach. Kylhammar and colleagues showed in 530 incident patients that reaching low risk at early follow-up, after a median of about four months, independently predicted long-term survival, and that survival was similar whether patients stayed low-risk or improved to it [3]. A later longitudinal analysis found that most patients never met low-risk criteria during three years and that the category reached at one year largely set the later course. Of patients who were low-risk at one year, 66% remained so at three years, whereas only 12% of those at intermediate risk and none at high risk reached low risk later. The pattern was more pronounced in older patients with more comorbidities, only 8% of whom were low-risk at baseline [50]. Two national cohorts tested whether this approach carries over to other settings. The Chinese registry of 2,031 patients, where CHD-PAH was the most common cause at 45.2%, supported the use of the main risk tools and described how goal-oriented care changed over time [20]. In Finland, the 28% of patients who remained at intermediate-high or high risk at one year were older and had more comorbidities, and low-risk attainment improved over calendar time as dual therapy became more common [51]. Both patient characteristics and incomplete use of guideline-based escalation therefore seem to contribute when patients fail to reach low risk.

Sotatercept

Four reports evaluated sotatercept, an activin-signalling inhibitor, in every case added to established background therapy. In STELLAR, which enrolled mainly WHO-FC II and III patients on dual or triple therapy, sotatercept improved 6MWD by about 41 m over placebo at week 24, improved eight of nine secondary endpoints, and reduced a post hoc composite of death or clinical worsening by about 84% [9]. Its haemodynamic sub-study confirmed lower mean pulmonary arterial pressure and PVR and better right heart function [31]. ZENITH enrolled high-risk patients with WHO-FC III or IV symptoms and a REVEAL Lite 2 score of 9 or more on maximum tolerated therapy. Sotatercept reduced the composite of death, lung transplantation, or PAH hospitalisation by 76% (HR 0.24, 95% CI 0.13 to 0.43; 17.4% versus 54.7%), and the trial was stopped early for efficacy [10]. In the SOTERIA extension, 426 participants were followed for a mean of about 449 days; improvements in 6MWD, NT-proBNP, WHO-FC, and simplified French risk score were largely maintained at one year [32]. A single-centre study of 45 patients with cardiopulmonary comorbidities, who would have been excluded from the trials, reported lower oxygen requirements, a 6MWD gain of about 20 m, and better REVEAL Lite 2 scores at six months [60]. Benefit in these studies did not depend on the background regimen.

Comparison of Risk-Stratification Tools

Low-risk definitions were grouped by tool type rather than assumed quality: Group 1 comprised the ESC/ERS three- and four-strata models; Group 2, multivariable prediction tools (COMPERA, COMPERA 2.0, REVEAL 2.0, REVEAL Lite 2, and Pulmonary Hypertension Outcomes Risk Assessment (PHORA)); and Group 3, the simplified French non-invasive criteria and French risk score. Table 6 summarises the operational definitions of low-risk status across the frameworks used in the included reports, while Table 7 compares the contemporary risk-stratification frameworks in terms of their classification structure, variables, prognostic discrimination, and practical clinical utility. The same patients could be classified differently depending on the tool used. In the Italian upfront-combination cohort, 34.8% were classified as low risk by REVEAL compared with 43.1% using the ESC/ERS approach at the same time point [45]. Four-strata models generally separated survival better than three-strata models, particularly at follow-up [36,38,49]. REVEAL 2.0 showed greater discrimination for one-year mortality than COMPERA and the French three-strata approach in its derivation cohort (C-statistic 0.76 vs 0.62 and 0.64) [4] and was subsequently validated in the Australian and New Zealand registry [13]; REVEAL Lite 2 achieved similar discrimination using six variables [5]. Only 5.6% of patients were classified as low risk at baseline using COMPERA 2.0 [6], while the French four-strata model showed better follow-up discrimination than the three-strata model (Harrell’s C 0.73 vs 0.69) [7]. Tools also differed in their response to treatment: although the direction of risk-category change was generally concordant, the magnitude of change and proportion reaching low risk varied [39,41]. COMPERA 2.0 and REVEAL Lite 2 showed close agreement in a Mexican cohort [19], whereas in CHD-PAH, both the French model and REVEAL Lite 2 were applicable to 126 patients despite lacking specific development or validation in this population [22]; supportive evidence was reported from a Chinese registry in which CHD-PAH predominated [20]. Overall, Table 7 highlights substantial heterogeneity in the number and type of variables, risk categories, prognostic performance, and practical applicability of contemporary risk-stratification frameworks, underscoring that these tools are not fully interchangeable across clinical settings.

**Table 6:** Operational definitions of low-risk status across the frameworks applied in the included reports 6MWD: six-minute walk distance; BNP: B-type natriuretic peptide; COMPERA: Comparative, Prospective Registry of Newly Initiated Therapies for Pulmonary Hypertension; ESC/ERS: European Society of Cardiology/European Respiratory Society; NT-proBNP: N-terminal pro-B-type natriuretic peptide; PHORA: Pulmonary Hypertension Outcomes Risk Assessment; REVEAL: Registry to Evaluate Early and Long-Term Pulmonary Arterial Hypertension Disease Management; SPAHR: Swedish Pulmonary Arterial Hypertension Registry; WHO-FC: World Health Organization functional class

Framework	Included reports applying it	Key parameters	Operational definition of low risk
ESC/ERS three-strata (SPAHR/COMPERA-derived)	Kylhammar 2018 [3]; Bouzina 2021 [50]; Boucly 2021 [44]; Badagliacca 2021 [45]; Badagliacca 2018 [48]; Quan 2022 [20]	Up to eight clinical, functional, biochemical, and haemodynamic variables	Mean risk score 1.0 to 1.49
ESC/ERS four-strata (non-invasive)	McLaughlin 2024 [30]; Pentikäinen 2025 [51]; Deshwal 2023 [55]	WHO-FC, 6MWD, BNP/NT-proBNP	Four-strata score <1.5
COMPERA 2.0	Hoeper 2025 [36]; Rosenkranz 2025 [38]; Rosenkranz 2023 [49]; Distler 2024 [21]; Tokgöz 2026 [54]; Cortes-Telles 2026 [19]; Vaidya 2024 [59]; Pentikäinen 2025 [51]	WHO-FC, 6MWD, BNP/NT-proBNP with refined cut-offs	Score 1.0 to 1.49
REVEAL 2.0	Badagliacca 2021 [45]; Fauvel 2023 [25]; Sitbon 2020 [27]; Tonelli 2020 [41]; Benza 2022 (FREEDOM-EV) [39]; D’Alto 2020 [47]; Deshwal 2023 [55]	13 weighted variables including non-modifiable prognostic factors	Score ≤6 (low <7 in cohort applications)
REVEAL Lite 2	Benza 2024 (GRIPHON) [28]; Benza 2022 (PATENT) [35]; Yaylalı 2023 [22]; Miller 2023 [42]; Gomez Rojas 2025 [60]; Tokgöz 2026 [54]; Cortes-Telles 2026 [19]; McLaughlin 2024 [30]	Six weighted, predominantly non-invasive variables	Score ≤5 (1–5 low, 6–7 intermediate, ≥8 high)
REVEAL (original)	Escribano-Subias 2020 [58]; D’Alto 2026 [57]	12 weighted components	Grouped strata, 1–7 low risk
PHORA	Fauvel 2023 [25]	Bayesian network model	Predicted one-year mortality <5%
French non-invasive criteria	Cui 2026 [52]; Yaylalı 2023 [22]; Sitbon 2020 [27]; Tonelli 2020 [41]; Benza 2022 (FREEDOM-EV) [39]; Zhao 2024 [53]; D’Alto 2026 [57]	WHO-FC, 6MWD, NT-proBNP	All three low-risk criteria met (WHO-FC I/II, 6MWD >440 m, NT-proBNP <300 pg/mL)
Simplified French risk score	Preston 2025 [32]	Count of favourable non-invasive criteria	Higher count indicates lower risk; not dichotomised
Goal-directed single-parameter targets (Category 3)	Benza 2018 [33]; Hoeper 2017 [34]; Simonneau 2020 [37]; Wang 2023 [43]; Lachant 2023 [61]	6MWD, WHO-FC, NT-proBNP thresholds or composite improvement	Threshold attainment, not multidimensional low-risk status
REVEAL Lite 2 high-risk entry criterion	Humbert 2025 [10]	Six-variable assessment	Score ≥9 indicates high risk; not a definition of low risk

**Table 7:** Comparison of the contemporary risk-stratification frameworks identified in this review Groups reflect instrument type, not an evidentiary hierarchy. Group 1: ESC/ERS-derived guideline models; Group 2: validated multidimensional prediction-model instruments; Group 3: validated simplified or non-invasive criteria. No group is assumed superior to another; see the Hierarchy of Low-Risk Definitions section for the comparison criteria used. 6MWD: six-minute walk distance; BNP: B-type natriuretic peptide; ESC/ERS: European Society of Cardiology/European Respiratory Society; NT-proBNP: N-terminal pro-B-type natriuretic peptide; PHORA: Pulmonary Hypertension Outcomes Risk Assessment; REVEAL: Registry to Evaluate Early and Long-Term Pulmonary Arterial Hypertension Disease Management; WHO-FC: World Health Organization functional class

Framework	Instrument group	Strata	Core variables	Reported discrimination	Main contribution to this review
ESC/ERS three-strata	Group 1	3: low, intermediate, high	Up to eight clinical, functional, biochemical, haemodynamic	Overall discrimination approximately 0.61 to 0.64, inconsistently reported	Established the multidimensional approach for baseline and follow-up assessment
ESC/ERS four-strata (non-invasive)	Group 1	4, by subdivision of intermediate risk	WHO-FC, 6MWD, BNP/NT-proBNP	Superior to three-strata at follow-up in registry and trial re-analyses	Supports the guideline recommendation for four-strata follow-up assessment
COMPERA 2.0	Group 2	4: low, intermediate-low, intermediate-high, high	WHO-FC, 6MWD, BNP/NT-proBNP with refined cut-offs	Outperforms three-strata assessment, particularly at follow-up	Improved discrimination within the heterogeneous intermediate-risk population
REVEAL 2.0	Group 2	Continuous score with derived categories	13 weighted demographic, clinical, functional, biochemical, haemodynamic variables	C-statistic approximately 0.76 for one-year mortality	Comprehensive model with strong discrimination; therapy-responsive in trial re-analyses
REVEAL Lite 2	Group 2	Continuous score with derived categories	Six weighted, predominantly non-invasive variables	C-index approximately 0.70 to 0.73 in the derivation cohort	Practical alternative to REVEAL 2.0; used as a trial-enrolment criterion in ZENITH
PHORA	Group 2	Predicted probability	Bayesian network of clinical variables	Not directly compared within the included reports	Alternative probabilistic classification used to define a low-risk subgroup
French non-invasive criteria / simplified French risk score	Group 3	Count of favourable criteria	WHO-FC, 6MWD, BNP/NT-proBNP	Prognostic at baseline and follow-up; three criteria associated with 94% lower event risk in GRIPHON	Simple, repeatable assessment applicable in routine and resource-limited settings

Secondary outcomes

Maintenance of Low Risk and Time to Attainment

Registry studies usually assessed risk at the first annual review [3,20,50,51], whereas trial analyses did so at 12 to 26 weeks [9,10,29,35,36]. Only one study measured time to improvement directly, reporting a median of 131 days and a cumulative improvement rate of 75.1% by 24 weeks with ambrisentan, with faster improvement in treatment-naive patients [43]. Using the French criteria, the proportion meeting all three rose from 12.3% to 50.7% with selexipag-containing triple therapy [52], and patients given selexipag in GRIPHON were more likely than those given placebo to gain criteria by week 26 [27]. Loss of low-risk status was common. Low-risk status was associated with better survival, transplant-free survival, and freedom from clinical worsening, whichever validated tool was used [3,20,27,28,35,44,50], but in SPAHR 27% of patients who were low-risk at one year had moved to intermediate or high risk by three years [50]. Four-strata assessment captures more of this movement because it splits the intermediate group [36,38]. The Turkish cohort suggested that early gains from sequential triple therapy may fade after one year [54], whereas gains with sotatercept were largely maintained at one year [32], and risk reduction after add-on triple therapy persisted over a median of 68 weeks [55].

Clinical, Functional, and Haemodynamic Outcomes

Clinical outcomes were the most frequently reported secondary outcomes and tracked closely with risk status [2,3,9,10,20,21,26,28,35,44,50]. The largest differences between strategies were in the French registry, with 91% five-year survival for parenteral triple therapy against 61% for monotherapy or dual therapy [44], and in the sotatercept trials [9,10]. Meeting the REPLACE composite endpoint in PATENT was linked to lower later mortality [37]. The 6MWD and functional class improved with almost every strategy studied. Examples include 6MWD values of 274, 308, and 327 m at baseline, week 16, and week 28 in COMPASS-3 [33]; gains of 55.0 m and 56.4 m with triple and dual therapy in TRITON [29]; an increase from 414 to 480 m, with the proportion in WHO-FC II rising from 26.7% to 65.8%, on selexipag-based regimens [52]; and a 31 m gain after switching to riociguat [34]. NT-proBNP fell alongside these changes by 74% and 75% in TRITON [29] and from 928 to 455 pg/mL in the Chinese selexipag cohort [52]. Actigraphy showed that total daily steps did not change after treatment was intensified, while a measure of peak activity capacity did increase [61]. Quality of life was reported as a separate outcome in only a few studies.

Haemodynamic outcomes came mostly from studies with routine right heart catheterisation. PVR fell similarly with triple and dual oral therapy in TRITON, without differences in mean pulmonary arterial pressure, cardiac index, or mixed venous oxygen saturation [29]. Sotatercept lowered mean pulmonary arterial pressure by about 13.9 mmHg and reduced PVR at 24 weeks [31]. Upfront dual therapy reduced PVR by a mean of 37% over two years in an Italian cohort [47] and by 50% to 55% in the pooled TRITON and REPAIR analysis [30], with smaller reductions as cardiac comorbidities increased [46]. In portopulmonary hypertension, upfront combination reduced PVR by 57% against 32% with monotherapy [23]. Only three studies reported cardiac imaging. Initial triple therapy with selexipag improved right atrial area, right ventricular diameter, and tricuspid annular plane systolic excursion (TAPSE) more than sequential addition, although full reversal of remodelling was uncommon [52]. Add-on triple therapy improved cardiopulmonary measures together with risk category [55], and a comparison of strategies found the greatest right ventricular improvement with a prostanoid combined with an oral drug [48].

Safety

Treatment escalation was not generally associated with excess harm. Discontinuation due to adverse events was more common with selexipag than placebo in GRIPHON (14.3% versus 7.1%) [26]. In TRITON, headache, diarrhoea, and nausea were the most common adverse events with triple therapy, and 16.0% stopped selexipag because of them compared with 14.2% stopping placebo [29]. In the Chinese selexipag cohort, prostacyclin-related side effects led a few patients to stop early, and cost was the most common reason for stopping overall [52]. In RESPITE, 52% of patients had drug-related adverse events and 16% had serious adverse events during the switch [34]. Epistaxis and telangiectasia were the most frequent adverse events of interest with long-term sotatercept [32]. The finding most relevant to this review came from the AMBITION post hoc analysis, in which low-risk patients stopped treatment because of adverse events somewhat more often with combination therapy than with monotherapy (13.0% versus 7.5% to 9.2%), although the difference was not significant [25].

Low-Risk Attainment by Treatment Strategy

Most reports gave the proportion of patients reaching low risk or a risk-category outcome from which it could be derived, and findings by strategy are summarised in Table 8.

**Table 8:** Evidence for low-risk attainment and clinically relevant outcomes according to treatment strategy Outcomes are reported as presented in the individual studies. Formal attainment of low-risk status using a validated multidimensional tool is distinguished from improvement in risk score or category, attainment of individual treatment targets, and clinical outcomes. Studies differed in population, design, comparator, risk framework and follow-up; therefore, findings should not be interpreted as direct comparisons of treatment effects across rows. Reports derived from overlapping parent studies or registries were considered together when synthesising the evidence. 6MWD: six-minute walk distance; CHD-PAH: congenital heart disease-associated pulmonary arterial hypertension; COMPERA: Comparative, Prospective Registry of Newly Initiated Therapies for Pulmonary Hypertension; ERA: endothelin receptor antagonist; ESC/ERS: European Society of Cardiology/European Respiratory Society; NT-proBNP: N-terminal pro-B-type natriuretic peptide; PAH: pulmonary arterial hypertension; PDE5i: phosphodiesterase type-5 inhibitor; PVR: pulmonary vascular resistance; REVEAL: Registry to Evaluate Early and Long-Term Pulmonary Arterial Hypertension Disease Management; WHO: World Health Organization

Study/report	Treatment strategy	Risk framework or outcome	Comparator	Main findings	Follow-up
Benza et al. 2018 (COMPASS-3) [33]	Bosentan, with escalation to sildenafil if required	Six-minute walk distance (6MWD); Category 3	Bosentan alone versus subsequent escalation	17% reached the prespecified 6MWD target on bosentan alone at week 16; a further 20% reached the target by week 28 after escalation.	16–28 weeks
Escribano-Subias et al. 2020 (PRACMA) [58]	Macitentan in routine practice	REVEAL score	Baseline status	Mean REVEAL score decreased by 1.4 points after treatment initiation.	Median approximately 10 months
Wang et al. 2023 [43]	Ambrisentan	Risk improvement	Baseline status; treatment-naïve versus add-on patients	Risk improvement was reported in 64.6% by 24 weeks, with faster improvement in treatment-naïve patients.	Up to 24 weeks
Galiè et al. 2015 (AMBITION) [2]	Upfront ambrisentan plus tadalafil	Clinical failure	Pooled monotherapy groups	Upfront combination therapy approximately halved the risk of clinical failure compared with either drug alone, mainly through fewer PAH-related hospitalisations.	Trial follow-up
Badagliacca et al. 2021 [45]	Upfront endothelin receptor antagonist (ERA) plus phosphodiesterase type-5 inhibitor (PDE5i)	REVEAL and ESC/ERS risk assessment	Baseline status	At six months, 34.8% were low-risk by REVEAL and 43.1% by ESC/ERS assessment; low-risk attainment was associated with haemodynamic response.	6 months
D’Alto et al. 2020 [47]	Upfront ambrisentan plus tadalafil	REVEAL 2.0; pulmonary vascular resistance (PVR)	Baseline status	Low-risk status increased from 9% at baseline to 43% at two years, although 57% remained at intermediate or high risk. Mean PVR decreased by 37%.	2 years
McLaughlin et al. 2024 [30]	Upfront macitentan plus tadalafil	Risk status and PVR	Baseline status; cardiac-comorbidity subgroups	Most patients improved their risk status by week 26. PVR decreased by approximately 55% in patients without cardiac comorbidities and 50% in those with one or two.	26 weeks
Boucly et al. 2021 [44]	Initial dual therapy versus initial triple therapy including parenteral prostacyclin	Low-risk criteria and survival	Initial monotherapy, dual therapy or triple therapy	Initial triple therapy including parenteral prostacyclin was associated with higher five-year survival. Reported low-risk attainment estimates require cautious interpretation because of inconsistencies in the source report.	Risk assessment: median 5 months; survival: 5 years
Distler et al. 2024 [21]	Initial ERA plus PDE5i in connective-tissue-disease-associated PAH	Survival	Initial monotherapy	Initial combination therapy was associated with better survival in systemic-sclerosis-associated PAH.	See original report
Cui et al. 2026 [52]	Selexipag-containing triple therapy	French non-invasive low-risk criteria	Initial triple therapy versus sequential addition of selexipag	All three French low-risk criteria were met by 81.5% versus 44.3%, respectively. The progression finding was exploratory because only six events occurred.	See original report
Zhao et al. 2024 [53]	Sequential oral triple therapy including selexipag	Feasibility and clinical response	Baseline status	A multicentre Chinese cohort supported the feasibility of adding selexipag to ERA plus PDE5i or riociguat therapy.	6 months
Deshwal et al. 2023 [55]	Add-on triple therapy in patients not at treatment goal	Low-risk attainment	Baseline status before third-drug addition	The proportion at low risk increased from 38.2% to 61.8%; no patients remained high-risk at the reported follow-up.	Median 68 weeks
Tokgöz et al. 2026 [54]	Sequential triple therapy including selexipag	COMPERA 2.0, ESC/ERS and REVEAL Lite 2	Baseline and longitudinal assessments	Early risk improvement was observed, but the effect appeared to wane after the first year. Early reclassification did not reliably discriminate long-term survival.	Beyond 1 year
Cortes-Telles et al. 2026 [56]	Add-on selexipag (RAMPHA cohort)	Low-risk attainment	Baseline status	The proportion classified as low-risk approximately tripled at six to twelve months, although the overall change was not statistically significant.	6–12 months
D’Alto et al. 2026 [57]	Macitentan, selexipag or both	Non-invasive low-risk criteria	Longitudinal assessment within the study	The mean number of non-invasive low-risk criteria changed little over 12 months.	12 months
Hoeper et al. 2017 (RESPITE) [34]	Switch from PDE5i to riociguat	6MWD, WHO functional class and NT-proBNP	Baseline status before switching	Mean 6MWD increased by 31 m; NT-proBNP decreased by 347 pg/mL; functional class improved in 54%. Clinical worsening occurred in 10%.	24 weeks
Benza et al. 2022 (PATENT) [35]	Riociguat	REVEAL Lite 2 score and risk stratum	Riociguat versus placebo	Riociguat improved risk score and risk stratum more often than placebo. Baseline score and score change were associated with subsequent outcomes.	See original report
Chin et al. 2021 (TRITON) [29]	Initial oral triple therapy	PVR, disease progression and mortality	Initial dual therapy	The primary endpoint was not met: PVR decreased by 54% with triple therapy and 52% with dual therapy. Exploratory findings for progression and mortality were not definitive.	26 weeks
Kiko et al. 2024 [23]	Upfront combination therapy in portopulmonary hypertension	PVR and event-free survival	Combination therapy versus monotherapy	PVR decreased by 57% versus 32%; event-free survival favoured combination therapy, with no detectable mortality difference.	See original report
Miller et al. 2023 (EXPEDITE) [42]	Parenteral treprostinil followed by oral treprostinil	REVEAL Lite 2 and target-dose attainment	Baseline status	79% reached the target oral dose; median REVEAL Lite 2 score decreased from 6 to 3.5.	16 weeks
Sitbon et al. 2015 (GRIPHON) [26]	Selexipag added to background therapy	First morbidity or mortality event	Selexipag versus placebo on background therapy	Selexipag reduced the risk of a first morbidity or mortality event by 40%.	Event-driven
Sitbon et al. 2020 [27]	Selexipag added to background therapy	Low-risk criteria and REVEAL 2.0	Selexipag versus placebo	Selexipag-treated patients were more likely to gain low-risk criteria by week 26; baseline criteria and REVEAL category were prognostic.	26 weeks
Benza et al. 2024 [28]	Selexipag added to background therapy	REVEAL Lite 2	Selexipag versus placebo	Risk improvement favoured selexipag across baseline risk categories. Mediation analysis suggested that risk-status improvement accounted for part of the treatment effect.	16–52 weeks
Benza et al. 2022 (FREEDOM-EV) [39]	Oral treprostinil	Risk score and clinical worsening	Oral treprostinil versus placebo	Risk scores predicted clinical worsening and were responsive to treatment.	See original report
Tonelli et al. 2020 [41]	Inhaled treprostinil	REVEAL 2.0 risk stratum	Inhaled treprostinil versus placebo	Improvement in REVEAL 2.0 risk stratum favoured inhaled treprostinil at week 12.	12 weeks

Treatment Benefit by Baseline Risk

In the AMBITION post hoc analysis, patients classified as low-risk by REVEAL 2.0 or PHORA did not have significantly less clinical worsening with upfront combination therapy than with monotherapy at one or three years. Once adverse events were added to clinical worsening in a combined benefit-risk endpoint, combination therapy no longer came out ahead in this group. For intermediate- and high-risk patients, benefit remained significant after accounting for adverse events (one-year HR 0.34, p=0.002; three-year HR 0.59, p=0.02, by REVEAL 2.0) [25]. This finding is at odds with the GRIPHON re-analyses. Using REVEAL Lite 2, selexipag reduced events in every baseline category, including low risk (HR 0.573, 95% CI 0.361 to 0.908, p=0.0178) [28], and the earlier analysis found a similar treatment effect regardless of the number of low-risk criteria [27]. The French registry found survival benefit from more intensive initial therapy in intermediate-risk patients [44]. When intermediate-low and intermediate-high patients were compared directly in PATENT, relative improvement was similar (38% versus 42%) [36], suggesting that this split may matter more for prognosis than for predicting treatment response. Baseline risk profiles also varied widely between cohorts: 9% of the Italian upfront-combination cohort was low-risk by REVEAL 2.0 at baseline [47], 3% of United States patients starting parenteral prostacyclin were low-risk by COMPERA 2.0 [59], and 35% of the Finnish cohort was low-risk at one year [51]. Given the conflicting results, whether treatment benefit depends on baseline risk remains an open question.

Subgroup Findings

These subgroup analyses were planned as descriptive and should be read as hypothesis-generating. Evidence by PAH phenotype came mostly from single studies with very low certainty. In COMPERA, systemic sclerosis-associated PAH carried the highest mortality and lupus-associated PAH the lowest, and the survival benefit of initial combination therapy was confined to the systemic sclerosis group [21]. The portopulmonary cohort was too small to detect a mortality difference and did not fully account for liver disease severity, with 82% of patients in Child-Pugh class A [23]. A Mexican cohort restricted to associated PAH (Group 1.4) found clear improvement in risk classification with sequential ERA and PDE5i therapy [19]. In a pan-African cohort, HIV-associated PAH behaved as a distinct high-risk phenotype, with worse three-year survival than pulmonary hypertension of other causes among people living with HIV, in patients younger than typical European or North American cohorts and with high rates of previous tuberculosis [24].

Age and comorbidity affected both the chance of reaching low risk and the meaning of a given risk category, and this was one of the most reproducible findings in the review. In SPAHR, only 8% of patients aged over 75 years or with three or more comorbidities were low-risk at baseline, and few reached low risk later [50]. In COMPERA, improvements in functional class, 6MWD, natriuretic peptides, and four-strata category were smaller in idiopathic PAH patients with any comorbidity, although the four-strata tool still predicted outcome in this group [49]. The Italian cohort showed the haemodynamic counterpart, with smaller falls in PVR as comorbidities accumulated [46]. In pooled riociguat trials, 6MWD and NT-proBNP improved regardless of comorbidity, but WHO-FC and COMPERA 2.0 category improved only in patients with none or one to two comorbidities [38]. Macitentan and tadalafil were effective in patients with one or two cardiac comorbidities [30], patients who failed to reach low risk in Finland were older and more comorbid [51], and real-world sotatercept data extended benefit to comorbid patients excluded from the trials [60].

Follow-up duration shaped what each study could show. Studies of six months or less, largely trial analyses and short-term cohorts [19,29-31,33,35-37,41-43,45,53], showed functional, biomarker, haemodynamic, and early risk improvement but were too short to assess hard outcomes or durability. Studies of six to 12 months [9,10,25,28,52,56-58] showed risk-category change and treatment benefit, mainly in intermediate- and high-risk patients. Studies longer than 12 months [3,20,21,24,32,44,47,49-51,54,55] provided most of the evidence on durability and long-term prognosis. By tool, ESC/ERS models were used most often across strategies and in registries; REVEAL and COMPERA tools were favoured in trial re-analyses and served as an enrolment criterion in ZENITH [10]; and the French criteria appeared mainly in trial re-analyses and in Asian and Turkish cohorts [22,27,39,41,52,53]. Studies that applied more than one tool to the same patients were the most useful for judging agreement between tools [19,39,41,54].

Factors Associated With Reaching Low Risk

Six factors recurred across studies. Older age and more comorbidities lowered both the baseline likelihood of low risk and the improvement achieved with treatment [46,49-51]. PAH aetiology mattered: systemic sclerosis-associated PAH, portopulmonary hypertension, and HIV-associated PAH carried worse risk profiles or survival than idiopathic or heritable disease [21,23,24]. Failure to reach low risk by the first annual review strongly predicted continued failure [50,54]. The size of the haemodynamic response was closely linked to reaching low risk, and many patients stayed above the threshold despite a clear fall in PVR after upfront combination therapy [45,47]. Regimens including a prostacyclin-pathway drug, particularly a parenteral one, were most often associated with risk reduction and survival benefit, while monotherapy and, to a lesser degree, oral dual therapy were least often associated with reaching low risk [26,33,42,44,55]. Finally, adverse events and cost limited sustained treatment, as shown by discontinuations with prostacyclin-pathway drugs [26,29], stopping because of cost [52], and more discontinuations with combination therapy among low-risk AMBITION patients [25].

Sensitivity Analyses

Several sensitivity analyses were undertaken to examine the robustness of the review findings. Because risk-of-bias assessments used different tools and classification systems, the judgements were interpreted according to the criteria of the respective tools rather than combined into a single overall scale. Some studies were judged to have a serious risk of bias under ROBINS-I or high risk-of-bias concerns under QUIPS (Table 9). Therefore, the previous statement that no included study had a high or critical overall risk of bias has been removed. The planned sensitivity analysis excluding studies at high or critical risk of bias was not conducted as originally intended, and this deviation is acknowledged.

**Table 9:** Summary of risk-of-bias assessments across the 48 included reports Detailed study-level, domain-specific risk-of-bias judgements and supporting rationales are provided in Appendix 1. The overall classifications should be interpreted according to the criteria of the respective assessment tools. RoB 2: Cochrane Risk of Bias 2 tool; ROBINS-I: Risk Of Bias In Non-randomized Studies of Interventions; QUIPS: Quality In Prognosis Studies

Evidence group	Risk-of-bias tool	Reports, n	Overall judgement	Principal sources of bias or concern
Primary randomised controlled trials	RoB 2	5	Low risk overall	Some concerns regarding outcome measurement or selection of reported results where secondary, exploratory, or post hoc endpoints were considered; one trial was stopped early for efficacy.
Prospective single-arm and open-label extension studies	ROBINS-I (pre-treatment comparator)	4	Moderate to serious	Absence of a control arm; regression to the mean in inadequate-responder populations; survivor selection into open-label extension studies.
Prospective non-randomised interventional study	ROBINS-I	1	Moderate	Open-label design and single-arm comparison for risk-related outcomes.
Secondary and post hoc analyses of trial data	RoB 2 applied to parent trial plus outcome-specific appraisal	12	Some concerns	Post hoc subgroup analyses; secondary composite outcomes; repeated analyses of the same parent trials; modelling assumptions in mediation analyses.
Large registry-based treatment-strategy studies	ROBINS-I	6	Moderate	Confounding by indication; differences in baseline disease severity, age, comorbidity, and treatment selection.
Longitudinal registry and national cohorts	ROBINS-I and/or QUIPS according to study objective	6	Moderate	Attrition; incomplete follow-up; variation in timing of reassessment; changes in clinical practice over calendar time.
Multicentre and single-centre observational cohorts	ROBINS-I	10	Moderate to serious	Residual confounding; absence of matching or adequate adjustment; variable completeness of risk-variable ascertainment.
Small phenotype-specific observational cohorts	ROBINS-I and/or QUIPS according to study objective	4	Moderate to serious	Small sample sizes; residual confounding; non-standardised ascertainment; phenotype-specific prognostic factors not captured by standard risk tools.

Removing the 12 secondary and post hoc analyses did not change the association between low-risk status and better outcomes. However, it removed much of the evidence on how individual risk tools respond to treatment and all direct evidence addressing whether treatment benefit depends on baseline risk. Removing the four single-arm studies [32-34,42] left the conclusions regarding low-risk attainment by treatment strategy unchanged but removed almost all evidence on treatment switching and rapid parenteral induction, indicating that these strategies are supported predominantly by uncontrolled data.

Excluding Category 3 evidence [33,34,37,43,61,62] did not change the findings, as all remaining studies used a recognised risk-assessment tool. Restricting the analysis to one report per overlapping population did not alter the overall conclusions, although limiting the riociguat evidence to one report per parent trial made the evidence for treatment switching appear less extensive. Removing the four small phenotype-specific studies [19,22-24] left the general conclusions unchanged but substantially reduced the evidence available for specific phenotypes.

Finally, restricting the analysis to studies published from 2020 onwards did not change the direction of the main findings. However, this restriction excluded the foundational treat-to-target study [3] and landmark trials of upfront dual therapy and selexipag [2,26], on which much of the more recent evidence builds.

Risk of Bias and Certainty of Evidence

Risk of bias was assessed for all 48 included reports using the tool appropriate to the study design and objective. The five primary RCTs (AMBITION, GRIPHON, TRITON, STELLAR, and ZENITH) were assessed using RoB 2, with detailed study-level judgements provided in Appendix 1. Concerns relating to the interpretation of selected outcomes included the use of exploratory or post hoc analyses, secondary composite endpoints, and the potential effect of early trial termination. In TRITON, the disease-progression findings required cautious interpretation because they arose from an exploratory analysis [29]. Similarly, the early termination of ZENITH for efficacy may have led to an overestimation of the treatment effect [10]. The 12 secondary and post hoc analyses also raised concerns because some risk-stratified analyses were not prespecified, multiple publications drew on the same parent trials, and mediation analyses relied on assumptions that could not be directly verified [25,27,28,35-39,41]. Detailed assessments of the methodological concerns affecting the secondary and post-hoc analyses are provided in Appendix 4.

The single-arm and open-label studies assessed using ROBINS-I had moderate to serious risk-of-bias concerns, principally because they lacked concurrent control groups, were susceptible to regression to the mean among inadequate responders, and, in the open-label extension setting, could be affected by selective retention of patients who had tolerated the parent trial [32-34,42]. These design limitations restrict causal interpretation of observed changes in risk status and do not permit reliable conclusions about comparative treatment effectiveness. Registry-based and observational treatment comparisons also showed moderate to serious risk-of-bias concerns, particularly because of confounding by indication and baseline differences between treatment groups. In the French registry, patients receiving triple therapy were younger and had fewer comorbidities despite worse haemodynamics [44]. Similar, although smaller, baseline imbalances were observed in the Chinese selexipag [52] and portopulmonary hypertension [23] cohorts. Although propensity-score matching was used in the French registry [44], confounding remained a concern in several smaller studies without comparable adjustment [19,22,23,52,56]. Missing-data handling appeared adequate in the larger registries, although reporting was less clear in several small single-centre cohorts [19,20,22,23,49-52,56]. ROBINS-I assessments of single-arm and open-label studies are discussed in Appendix 3. Detailed ROBINS-I assessments of the registry-based and observational studies are provided in Appendix 5.

For studies assessed using QUIPS, the SPAHR studies and the Chinese registry were judged to have low risk of bias in the domains of study participation, attrition, and prognostic-factor measurement [3,20,50]. In contrast, several small phenotype-specific cohorts raised concerns regarding outcome measurement and control of confounding, including liver disease severity and previous tuberculosis [19,22-24]. Some non-randomised studies received serious risk-of-bias judgements under ROBINS-I, while some prognostic studies were assessed as having high risk-of-bias concerns under QUIPS. QUIPS assessments of prognostic studies are discussed in Appendix 2. These classifications are specific to their respective assessment tools and should not be interpreted as directly equivalent to the RoB 2 categories. The summary of assessments across the 48 reports is presented in Table 9, and detailed study-level and domain-specific judgements, with supporting rationales, are provided in Appendix 1.

The certainty of evidence was considered separately from risk of bias, taking into account the design and consistency of the available evidence, imprecision, indirectness, and other relevant limitations. Accordingly, conclusions based predominantly on observational or uncontrolled studies were interpreted cautiously, particularly where residual confounding or the absence of a concurrent comparator limited causal inference.

Certainty of Evidence

Certainty was moderate that reaching or maintaining low risk is associated with better survival and freedom from clinical worsening. This rating concerns a prognostic association and does not mean that lowering a patient’s risk category causes better survival. Certainty was low to moderate for the advantage of upfront triple over dual therapy, because the only randomised comparison was imprecise [29] and the observational data were confounded [23,44,52]. Certainty was moderate that prostacyclin-pathway escalation improves risk status [26-28,39,41,42], but low to moderate that it reduces mortality outside parenteral regimens. Certainty was moderate that add-on sotatercept improves clinical outcomes, downgraded because patients were all on background therapy and the high-risk trial stopped early [9,10,32]. Certainty was very low that low-risk patients gain less from further treatment, because the evidence comes from one unreplicated post hoc analysis [25] and is contradicted by analyses of a different drug [27,28]. Certainty was moderate that four-strata models discriminate better than three-strata models and that REVEAL tools discriminate well [35,36,38,49], and very low for phenotype-specific findings [19,22-24]. Table 10 summarises these ratings.

**Table 10:** Certainty of evidence for clinically important outcomes associated with low-risk attainment and treatment strategy CHD-PAH: congenital heart disease-associated pulmonary arterial hypertension; ESC/ERS: European Society of Cardiology/European Respiratory Society; GRADE: Grading of Recommendations Assessment, Development and Evaluation; HIV: human immunodeficiency virus; PAH: pulmonary arterial hypertension; REVEAL: Registry to Evaluate Early and Long-Term Pulmonary Arterial Hypertension Disease Management

Clinical question / outcome	Reports contributing (n)	Overall finding	Risk of bias	Inconsistency	Indirectness	Imprecision	Publication bias	Overall certainty
Does achieving or maintaining low-risk status predict better clinical outcomes?	12	Low-risk status was consistently associated with improved survival and greater freedom from clinical worsening. This is a prognostic association and does not establish that treatment-induced risk reduction causes better outcomes.	Not downgraded in current assessment	Not downgraded in current assessment	Variation in populations and risk instruments	Not downgraded in current assessment	No downgrade documented	Moderate
Does upfront triple therapy provide greater benefit than upfront dual therapy?	4	Findings favoured regimens incorporating parenteral prostacyclin more consistently than oral triple therapy. The randomised oral comparison was imprecise, while observational comparisons were susceptible to confounding.	Concerns in observational evidence	Not downgraded in current assessment	Differences between regimens and populations	Concerns due to imprecise evidence	No downgrade documented	Low
Does prostacyclin-pathway intensification improve risk status?	7	Risk scores or categories generally improved across oral, inhaled and parenteral treatment pathways. Several reports derived from overlapping parent trials.	Not downgraded in current assessment	Not downgraded in current assessment	Not downgraded in current assessment	Not downgraded in current assessment	No downgrade documented	Moderate
Does prostacyclin-pathway intensification reduce mortality?	Subset of the above evidence	Mortality benefit was less certain outside parenteral prostacyclin-based regimens.	Concerns in non-randomised evidence	Not downgraded in current assessment	Differences in treatment pathways	Concerns due to imprecise evidence	No downgrade documented	Low
Do low-risk patients benefit less from further treatment intensification?	3	One post hoc analysis suggested attenuated benefit and possible excess treatment-related adverse events, whereas analyses of a different drug suggested benefit across risk categories.	Concerns related to post hoc evidence	Inconsistent findings across interventions	Not downgraded in current assessment	Concerns due to limited evidence	No downgrade documented	Very low
Do four-strata risk models perform better than three-strata models?	4	Four-strata models showed improved prognostic discrimination, particularly in separating intermediate-risk patients.	Not downgraded in current assessment	Not downgraded in current assessment	Concerns about cohort-specific calibration	Not downgraded in current assessment	No downgrade documented	Moderate
Do REVEAL-based instruments outperform unweighted ESC/ERS-derived approaches?	4	REVEAL-based instruments showed good prognostic discrimination in selected comparative cohorts.	Not downgraded in current assessment	Not downgraded in current assessment	Limited number of direct head-to-head comparisons	Not downgraded in current assessment	No downgrade documented	Moderate
Does add-on sotatercept improve outcomes compared with background therapy alone?	4	Add-on sotatercept was associated with improvements in functional, haemodynamic, risk and composite clinical outcomes. Evidence included randomised trials and an uncontrolled real-world cohort.	Reassess against study-level assessments	Reassess against outcome-specific findings	Background PAH therapy alone is not a reason to downgrade	Reassess precision and effect of early trial termination	No downgrade documented	Moderate (provisional)
Is treatment response established in specific PAH phenotypes?	5	Evidence was insufficient to draw firm conclusions for porto-pulmonary hypertension, congenital heart disease-associated PAH, Group 1.4 PAH or HIV-associated PAH.	Concerns where applicable	Not reliably assessable across single-study phenotypes	Concerns due to limited phenotype-specific evidence	Serious concerns due to small samples	No downgrade documented	Very low

Discussion

Principal Findings

Three main findings emerged from this review. Most patients did not achieve a fully low-risk profile with any treatment strategy, with fewer than half reaching low risk even after guideline-based upfront combination therapy in most cohorts [30,45,47,51]. Strategies incorporating prostacyclin-pathway therapy, particularly parenteral regimens, were associated with improvements in risk status and survival, while add-on sotatercept improved clinical and haemodynamic outcomes in patients who remained above the low-risk threshold despite established therapy [9,10,26,31,32,42,44]. Low-risk status, once achieved, was only moderately durable, and risk category at the first annual review was strongly associated with subsequent clinical course [3,50]. These findings support early reassessment and timely treatment escalation when low-risk status is not achieved.

Whether patients already classified as low risk derive meaningful benefit from further treatment intensification remains less certain. A post hoc analysis of AMBITION found no significant benefit and a possible excess of treatment-related adverse events in this subgroup [25], whereas re-analyses of GRIPHON demonstrated a treatment benefit with selexipag across baseline risk categories, including low-risk patients [27,28]. This inconsistency highlights that low-risk status should not yet be regarded as a validated surrogate endpoint. In GRIPHON, mediation analyses indicated that changes in risk score explained approximately one-fifth of the overall treatment effect on morbidity and mortality, increasing to nearly half among patients with higher baseline risk [28]. An individual-patient analysis across three randomised PAH trials also found that low-risk status did not fully satisfy statistical criteria for surrogate validity [63]. Low-risk status should therefore be regarded as an important treatment target and prognostic marker, but not as a substitute for hard clinical endpoints in future trials.

Treatment Strategies and Barriers to Reaching Low Risk

The evidence by strategy follows a clear gradient. Most patients on monotherapy do not reach a lasting low-risk category, and escalation within the first one to two years is the rule [33,43,51,58]. Upfront dual oral therapy improves functional class and exercise capacity reliably, but mean PVR reductions of 37% to 55% across cohorts and trials still leave the majority of patients above the low-risk threshold at six months to two years [30,45,47]. The only randomised comparison of initial oral triple versus dual therapy, TRITON, did not meet its primary haemodynamic endpoint, and any mortality signal was exploratory [29]. Registry data for triple therapy including a parenteral prostacyclin show substantially better five-year survival, around 91% compared with 61% for monotherapy or dual therapy in the French registry [44], but this comparison is confounded: the triple-therapy cohort was younger, less comorbid, and haemodynamically more severe, and propensity matching only partly corrects for this. For patients who do not reach low risk on initial therapy, adding a prostacyclin-pathway drug is well supported by randomised evidence for reducing disease progression [26,39,41], and parenteral escalation in high-risk patients has its strongest basis in survival registry data [44]. Sotatercept provides an additional add-on option, supported by randomised evidence for improving clinical outcomes in patients receiving established background therapy, with early real-world data extending this evidence to patients with cardiopulmonary comorbidities [9,10,32,60]

Several factors recurred across studies as predictors of failure to reach low risk. Older age and comorbidity burden limited both haemodynamic response and the prognostic signal of conventional risk tools [46,49-51]. PAH aetiology had independent effects, with systemic sclerosis-associated PAH, porto-pulmonary hypertension, and HIV-associated PAH each carrying worse profiles than idiopathic disease [21,23,24]. Failure at the first annual review strongly predicted continued failure [50,54]. Large haemodynamic response was the proximate determinant of attainment, and many patients remained above the threshold despite clear PVR reductions [45,47]. Treatment inertia and cost compounded these barriers: parenteral prostacyclin was initiated mostly when patients were already at intermediate-high risk in a large practice survey [59], and economic discontinuation affected prostacyclin use in resource-limited settings [52].

Durability, Risk Tools, and Instrument Choice

Low-risk status was not consistently maintained once achieved. In the longitudinal SPAHR cohort, 27% of patients who were low risk at one year had deteriorated by year three, whereas patients who remained above low risk at one year rarely subsequently reached the low-risk category [50]. This finding highlights the importance of the first annual assessment in subsequent treatment decisions and supports closer reassessment of patients who remain above low risk or who have recently undergone treatment changes [50,54]. Four-strata models, which subdivide the intermediate-risk category, provided better survival discrimination than three-strata models in registry and trial re-analyses [36,38,49]. REVEAL-based instruments also demonstrated strong discrimination and responsiveness to treatment-related changes in trial populations, although their application may require variables that are not routinely available in all clinical settings [28,35,39,41]. The simplified French three-criterion approach provides a more practical assessment based on fewer variables, although it may offer less discrimination than more granular four-strata approaches [7,27]. Consistent use of the same risk instrument at follow-up may facilitate interpretation of changes in risk status over time.

Several Group 1 PAH subtypes also have limitations in relation to existing risk tools. In CHD-PAH, the French model and REVEAL Lite 2 were not specifically derived for this population [22]. In porto-pulmonary hypertension, liver disease severity provides prognostic information beyond pulmonary haemodynamic measures, but this is not incorporated into conventional PAH risk scores [23]. In HIV-associated PAH, particularly in African cohorts, outcomes were influenced by HIV-related mortality and previous tuberculosis, factors that may not be adequately captured by standard PAH risk scores [24]. These findings do not preclude the use of risk stratification in these populations but indicate that risk scores should be interpreted alongside disease-specific and comorbidity-related factors [22-24].

Strengths and limitations

This review examined treatment strategies and low-risk attainment across multiple risk-stratification tools, patient populations, and study designs, including recent evidence on sotatercept, sequential triple therapy, and parenteral prostacyclin-based strategies. Design-appropriate risk-of-bias assessment was applied, overlapping populations were systematically tracked, and certainty of evidence was graded for the main conclusions. The literature search was conducted from database inception to August 2026 using four complementary bibliographic sources (PubMed/MEDLINE, Scopus, Dimensions, and the CENTRAL), with broad search concepts encompassing PAH, risk stratification, and treatment strategies. Although Embase was not searched, the combination of databases was designed to maximise retrieval across biomedical, multidisciplinary, clinical-trial, and citation-indexed literature and successfully identified the major randomised trials, registries, and contemporary observational studies underpinning the review. Nevertheless, Embase may identify additional biomedical and European literature, including records from journals and conference proceedings that may not be indexed in the other databases. Its absence may therefore have resulted in some eligible records being missed and should be regarded as a potential limitation of the search strategy rather than evidence that the overall literature search was non-comprehensive.

Several additional limitations should be considered. Clinical and methodological heterogeneity across the included studies limited direct comparison of treatment effects and precluded quantitative meta-analysis. Clinical heterogeneity arose from differences in PAH phenotype, baseline disease severity and risk, comorbidity burden, treatment strategy and sequence, background therapy, route of prostacyclin administration, and duration of follow-up. Methodological heterogeneity included differences in study design, including randomised trials, prospective and retrospective cohorts, registries, single-arm studies, and post hoc analyses, as well as differences in outcome definitions, timing of assessment, and risk-stratification instruments. These differences may influence the magnitude and clinical meaning of observed treatment-associated changes; therefore, findings should be interpreted primarily as patterns of evidence rather than as directly comparable estimates of comparative treatment effectiveness. Effect estimates were consequently derived from individual studies and should not be interpreted as pooled estimates.

The 48 reports represented a smaller number of underlying study populations, and multiple analyses from studies such as GRIPHON and PATENT should not be interpreted as independent replications. Differences between risk-stratification instruments may also affect the apparent proportion of patients achieving low-risk status, such that numerical differences between studies may partly reflect the instrument and thresholds used rather than differences in treatment efficacy. For example, in one included cohort, 34.8% of patients were classified as low risk using REVEAL compared with 43.1% using ESC/ERS criteria at the same time point, illustrating why proportions achieving low risk should not be compared across studies without considering the underlying instrument and its thresholds. Treatment strategies were likewise not assumed to be clinically equivalent. For example, “triple therapy” comprised different pharmacological combinations and routes of administration across studies; therefore, evidence for oral triple therapy could not be directly extrapolated to regimens incorporating parenteral prostacyclin. Low-risk attainment was a prespecified primary endpoint in relatively few included studies. Registry-based and other observational treatment comparisons are additionally susceptible to confounding by indication and baseline differences between treatment groups, while single-arm studies lack concurrent controls and therefore provide more limited evidence for causal treatment effects. Conversely, although randomised trials provide stronger evidence for treatment effects, their selected eligibility criteria may limit generalisability to routine clinical practice.

Although a written protocol was prepared before title and abstract screening began, it was not prospectively registered in PROSPERO (International Prospective Register of Systematic Reviews) or another public registry. Consequently, the protocol was not independently time-stamped or publicly available before the review commenced, limiting external verification of the prespecified methods and potentially reducing reproducibility. To mitigate this limitation, the review reports the protocol chronology, eligibility criteria, complete database-specific search strategies, search dates, study-selection procedures, and methodological amendments.

The evidence base was also concentrated in Western Europe, North America, and East Asia, which may limit generalisability to other healthcare settings and populations. Finally, quality-of-life and cardiac-imaging outcomes were infrequently reported across the included studies, limiting assessment of these clinically relevant dimensions of treatment response.

Implications for practice and research

Four practice implications are supported by the available evidence. Initial dual therapy frequently leaves patients above the low-risk threshold, supporting planned reassessment and consideration of treatment escalation when low risk is not achieved [30,45,47,51]. When further combination therapy is considered, the evidence differs according to prostacyclin-pathway strategy: oral and inhaled agents have randomised evidence supporting reductions in disease progression and improvements in risk status, whereas parenteral prostacyclin has stronger observational evidence for survival benefit [26,39,41,44]. For patients who remain above low risk despite established combination therapy, add-on sotatercept has demonstrated improvements in clinical and haemodynamic outcomes in randomised trials [9,10,32]. Failure to reach low risk by the first annual review was associated with a low likelihood of subsequent spontaneous improvement in the available longitudinal data [50,54]. These findings support systematic reassessment of treatment at this stage rather than assuming that risk status will improve without further intervention.

For research, several priorities emerge. Prospective trials should stratify or adjust analyses according to validated baseline risk categories to clarify the effects of treatment across different levels of baseline risk. Further work is also needed to evaluate whether changes in risk status fulfil accepted criteria for a surrogate endpoint, including formal analyses using established surrogate-validation frameworks [28,63]. Controlled studies are needed to better define the benefits and risks of rapid parenteral prostacyclin induction. Risk tools also require further validation in specific Group 1 PAH populations, including porto-pulmonary and HIV-associated PAH [23,24]. Finally, future trials should consider quality of life, cardiac imaging, and activity measures as prespecified outcomes, given their limited reporting across the current evidence base.

## Conclusions

Forty-eight reports spanning randomised trials, post hoc analyses, national registries, and observational cohorts in multiple countries support three conclusions. First, low-risk status is a meaningful but difficult treatment target. Most patients do not reach it even after guideline-concordant upfront combination therapy, and once reached, it is only moderately durable. Prostacyclin-pathway intensification and, as add-on therapy in patients not at goal, sotatercept showed the most consistent evidence for improving risk status and clinical outcomes, although the magnitude and mechanism of benefit differed by drug class and route of administration. Second, the risk category at the first annual review is the single strongest predictor of longer-term course, which argues for concentrating treatment decisions in the first year and for consistent use of a validated four-strata or REVEAL-based instrument at every follow-up visit. Third, low-risk status is not yet a validated surrogate endpoint. Improvement in risk scores explains only part of the treatment effect in mediation analyses, and the evidence on whether patients already at low risk benefit from further escalation remains conflicting and inconclusive. Managing the disease means aiming for low risk at every review, escalating when it is not reached, and recognising that older, comorbid patients and those with certain PAH phenotypes may face structural limits on how far standard tools can track their genuine clinical state.

## Appendices

Appendix 1

Risk-of-Bias Assessment of Included Studies

Risk of bias was assessed using RoB 2 for randomised controlled trials, ROBINS-I for non-randomised and observational studies, and QUIPS for prognostic studies. Two reviewers assessed studies independently, with disagreements resolved by consensus or third-reviewer adjudication. Risk-of-bias judgements were not converted into numerical scores.

**Table 11:** Randomised controlled trials (RoB 2) Key: L: low risk; SC: some concerns; H: high risk The original RoB 2 assessment used the five standard domains: randomisation, deviations from intended interventions, missing outcome data, outcome measurement, and selection of the reported result.

Study	Overall risk	Main concern
Galiè 2015 [2] (AMBITION)	SC	Post-hoc risk subgroup analysis; secondary composite endpoint
Sitbon 2015 [26] (GRIPHON)	SC	Post-hoc risk-stratified analysis; multiple analyses of the same trial
Chin 2021 [29] (TRITON)	SC	Exploratory disease-progression analysis; not powered for mortality
Hoeper 2023 [9] (STELLAR)	SC	Post-hoc composite endpoint used for risk analysis
Humbert 2025 [10] (ZENITH)	SC	Early termination for efficacy may overestimate effect size

Appendix 2

**Table 12:** Prognostic studies (QUIPS) Key: L: low; M: moderate; H: high concern The QUIPS assessment covered study participation, attrition, prognostic-factor measurement, outcome measurement, confounding, and statistical analysis/reporting.

Study	Overall risk	Main concern
Kylhammar 2018 [3]	L	Large incident SPAHR cohort; predefined assessment
Bouzina 2021 [50]	M	Attrition and calendar-time confounding
Quan 2022 [20]	M	Registry population differs from derivation cohorts
Pentikäinen 2025 [51]	M	Evolving treatment practice during the study period
Yaylalı 2023 [22]	H	Risk tools not validated specifically in CHD-PAH
Kiko 2024 [23]	H	Small sample; liver disease not captured
Thienemann 2022 [24]	H	HIV/TB competing risks
Cortes-Telles 2026 [19]	H	Very small, single-centre Group 1.4 cohort

Appendix 3

**Table 13:** Single-arm and open-label studies (ROBINS-I) Key: L: low; M: moderate; S: serious; C: critical

Study	Overall risk	Main concern
Benza 2018 [33] (COMPASS-3)	M	No control arm; regression to the mean
Hoeper 2017 [34] (RESPITE)	M	No control arm; selected patients with inadequate response
Miller 2023 [42] (EXPEDITE)	S	No control arm; selected patients; uncontrolled risk-score change
Preston 2025 [32] (SOTERIA)	M	Survivor selection into extension; no control group

Appendix 4

**Table 14:** Secondary and post-hoc trial analyses (overall judgement: some concerns for all studies) Key: L: low; M: moderate; S: serious; C: critical The principal concerns in these analyses were post-hoc subgroup analyses, outcome selection, and modelling assumptions.

Study	Parent trial	Main concern
Fauvel 2023 [25]	AMBITION	Post-hoc risk subgroup analysis
Sitbon 2020 [27]	GRIPHON	Post-hoc risk-criteria analysis
Benza 2024 [28]	GRIPHON	Mediation and modelling assumptions
Benza 2022 [35]	PATENT	REVEAL Lite 2 was not a primary endpoint
Hoeper 2025 [36]	PATENT-1/2	Retrospective application of COMPERA 2.0
Simonneau 2020 [37]	PATENT-1/2 / REPLACE	Retrospective composite outcome
Rosenkranz 2025 [38]	PATENT-1/2 + REPLACE	Pooled post-hoc analysis; small subgroups
Benza 2022 [39]	FREEDOM-EV	Risk scores were secondary analyses
Tonelli 2020 [41]	TRIUMPH + BEAT	Post-hoc pooled analysis
McLaughlin 2024 [30]	TRITON + REPAIR	Pooled analysis; small subgroups
Souza 2023 [31]	STELLAR	Haemodynamic sub-study; not a primary endpoint

Appendix 5

**Table 15:** Registry-based and observational studies (ROBINS-I) Key: L: low; M: moderate; S: serious; C: critical The original assessment identified confounding, selection, intervention classification, missing data, outcome measurement, and selective reporting as the principal ROBINS-I domains.

Study	Overall risk	Main concern
Boucly 2021 [44]	M	Residual confounding
Kylhammar 2018 [3]	M	Registry selection and timing of risk assessment
Bouzina 2021 [50]	M	Attrition and calendar-time effects
Quan 2022 [20]	M	Registry enrolment bias
Pentikäinen 2025 [51]	M	Cohort selection; incomplete capture of recent escalation
Distler 2024 [21]	M	Small CTD-PAH subgroups
Rosenkranz 2023 [49]	M	Comorbidity groups not matched
Badagliacca 2021 [45]	M	No comparator; treatment-selection bias
Badagliacca 2022 [46]	S	Unmatched comorbidity groups; small subgroups
D'Alto 2020 [47-alt]	M	Retrospective design; attrition
Badagliacca 2018 [48]	S	Small matched cohorts; residual confounding
Cui 2026 [52]	S	Few events; baseline imbalance
Zhao 2024 [53]	S	Retrospective design; mixed treatment backgrounds
Tokgöz 2026 [54]	M	Single-centre study; dose/tolerability limitations
Deshwal 2023 [55]	S	Retrospective single-centre study; treatment-selection bias
Bravo-Marques 2024 [56]	S	Very small sample; no control group
D'Alto 2026 [57]	M	Observational design; limited follow-up
Cortes-Telles 2026 [19]	S	Small single-centre cohort
Escribano-Subias 2020 [58]	M	Real-world study; limited risk-score data
Vaidya 2024 [59]	S	Chart review and clinician survey; possible selection bias
Gomez Rojas 2025 [60]	S	Small single-centre cohort
Lachant 2023 [61]	M	Small paired cohort; non-validated activity measure
Wang 2023 [43]	M	Potential confounding by indication
